# Epigenome engineering: new technologies for precision medicine

**DOI:** 10.1093/nar/gkaa1000

**Published:** 2020-11-16

**Authors:** Agustin Sgro, Pilar Blancafort

**Affiliations:** Cancer Epigenetics Laboratory, The Harry Perkins Institute of Medical Research, Nedlands, Western Australia 6009, Australia; School of Human Sciences, The University of Western Australia, Crawley, Perth, Western Australia 6009, Australia; Cancer Epigenetics Laboratory, The Harry Perkins Institute of Medical Research, Nedlands, Western Australia 6009, Australia; School of Human Sciences, The University of Western Australia, Crawley, Perth, Western Australia 6009, Australia; The Greehey Children's Cancer Research Institute, The University of Texas Health Science Center, San Antonio, TX 78229, USA

## Abstract

Chromatin adopts different configurations that are regulated by reversible covalent modifications, referred to as epigenetic marks. Epigenetic inhibitors have been approved for clinical use to restore epigenetic aberrations that result in silencing of tumor-suppressor genes, oncogene addictions, and enhancement of immune responses. However, these drugs suffer from major limitations, such as a lack of locus selectivity and potential toxicities. Technological advances have opened a new era of precision molecular medicine to reprogram cellular physiology. The locus-specificity of CRISPR/dCas9/12a to manipulate the epigenome is rapidly becoming a highly promising strategy for personalized medicine. This review focuses on new state-of-the-art epigenome editing approaches to modify the epigenome of neoplasms and other disease models towards a more ‘normal-like state’, having characteristics of normal tissue counterparts. We highlight biomolecular engineering methodologies to assemble, regulate, and deliver multiple epigenetic effectors that maximize the longevity of the therapeutic effect, and we discuss limitations of the platforms such as targeting efficiency and intracellular delivery for future clinical applications.

## INTRODUCTION

Maintenance of cellular identity requires faithful replication of the genome during cell division. However, the balance between ‘stability’ of the genetic information and ‘flexibility’ of the spatiotemporal control of gene expression is highly regulated in eukaryotic cells. Thus, while the genomic ‘infrastructure’ based on the nucleotide sequence is faithfully replicated during cell division, the genomic ‘superstructure’ at the chromatin level exhibits a high degree of conformational freedom (1). Such adaptability of chromatin states stems largely from causal chemical modifications of the DNA and its associated proteins. These ‘epigenetic marks’ modulate chromatin structure by regulating the accessibility of the DNA and the histones for binding to a multitude of proteins that orchestrate DNA replication, gene expression, and DNA damage responses (2). A multitude of cellular enzymes, or ‘writers’, are responsible for depositing specific marks on the DNA and at specific histone sites, while ‘erasers’ catalyze the specific removal of these covalent modifications (Supplementary Tables S1 and S2). Cells can, therefore, switch between distinct chromatin states ranging from ‘euchromatin’, characterized by accessible DNA, for actively transcribed genes, to the ‘heterochromatin’ of untranscribed genes, associated with a more condensed and inaccessible DNA, and gene silencing (3). Dysregulation of DNA and histone post-translational modifications has been linked with several diseases, including developmental and neurological disorders as well as neoplasms (4).

Due to the reversible nature of epigenetic modifications, ‘epidrugs’ have been clinically approved for manipulation of the epigenome. Epidrugs comprise specific inhibitors of DNA methyltransferases (DNMTs), histone-lysine methyltransferases (KMTs), histone-lysine acetyltransferase (KATs), histone-lysine demethylases (KDMs) and histone deacetylases (HDACs) (5). However, these inhibitors lack locus-selectivity and they potentially cause global changes in gene expression and toxicity in patients. To enhance the selectivity of epigenetic regulation, over the past two decades, artificial transcription factors (ATFs) have been engineered by fusion of a DNA-binding domain (DBD) to one or more effector domains (ED) to enable precise gene activation and repression at will (6).

The first ATFs were generated by linking zinc finger (ZF) modules, potentially targeting single genes by the incorporation of six ZFs that can bind to 18-base pair (bp) genomic sites (7). However, these platforms suffered from a high incidence of non-cognate site recognition when overexpressed in mammalian cells (8). This issue was circumvented by the development of transcription activator-like effectors (TALE) isolated from plant pathogenic bacteria of the *Xanthomonas* genus (9), which have demonstrated superior DNA selectivity than that of ZFs. Both ZF and TALEs have paved the way for the next generation of cutting-edge molecular tools that employ RNA-guided systems based on the Clustered Regularly Interspaced Short Palindromic Repeats/CRISPR-associated protein 9, referred to as CRISPR/Cas9. In this system, a 20-nucleotide (nt) guide RNA (gRNA) directs the Cas9 nuclease to cleave or nick DNA. The deactivated or catalytically dead version, referred to as CRISPR/dCas9 technology, has rapidly advanced the field of targeted gene regulation in recent years, due to its ease of targeting and its high versatility. Despite these advantages, CRISPR/dCas9 technology still suffers from significant impediments to its successful applicability.

In this review, we discuss the main molecular inducible and repressible systems that have been devised for epigenome engineering, with an emphasis on the new emerging technologies for combining effector domains (EDs) to maximize targeted epigenome engineering for applications in molecular precision medicine.

## EPIGENETIC EDITING TOOLS DERIVED FROM Cas9 AND Cas12a ORTHOLOGUES

**Figure 1.**
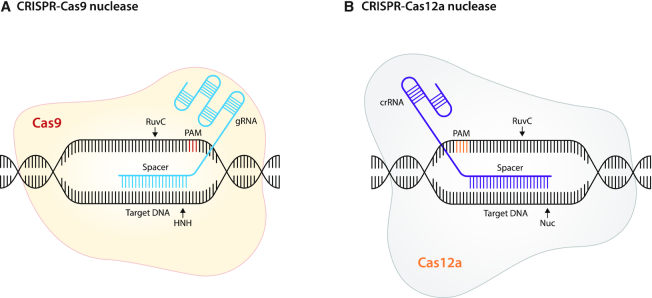
Schematic representation of the main CRISPR–Cas proteins adopted for epigenetic editing. **(A)** Cas9 proteins are RNA-guided DNA-targeting endonucleases. In epigenome engineering, the two Cas9 nuclease domains, RuvC and HNH, are mutated. Mutation of the catalytic residues of RuvC (D10A) and HNH (H840A) and (N580A) for *Streptococcus pyogenes* and *Staphylococcus aureus*, respectively, render Cas9 proteins defective, i.e., SpdCas9 and SadCas9. dCas9 proteins can still interact with the backbone of the guide RNA (gRNA). DNA binding results from complementary pairing of the spacer portion of the gRNA (20 nucleotides) to a targeted genomic region positioned next to a 5′ protospacer adjacent motif (PAM). **(B)** Cas12a endonucleases can be catalytically deactivated by point mutations. (D832A) in the RuvC domain and (E925A) in a putative nuclease (Nuc) domain render *Lachnospiraceae bacterium* Cas12a-defective; i.e., LbdCas12a, also known as LbdCpf1. DNA recognition and binding relies on the complementarity between the CRISPR RNA (crRNA) spacer (24 nucleotides), positioned next to a 3′ PAM, and the DNA target sequence.

The CRISPR–Cas system is part of an adaptive immunity mechanism present in a multitude of bacteria and archaea that encode a library of DNA fragments from foreign invaders, such as viruses and plasmids, for recognition and defense and ultimately destruction of foreign nucleic acids (10). All Cas proteins utilized in genome engineering rely on a guide RNA (gRNA) to target the enzyme in specific genomic sequences. The gRNA is a chimeric fusion of CRISPR RNA (crRNA) and its trans-activating CRISPR RNA (tracrRNA) for Cas9 (11), whereas for Cas12a it only consists of the crRNA (12). The gRNA comprises a ‘spacer’ of either 20 nucleotides (Cas9) or 24 nucleotides (Cas12a), which is the interchangeable portion of the gRNA complementary to the targeted genomic sequence and it is positioned next to a proto-spacer adjacent motif (PAM). The PAM-interacting domain of Cas proteins crucially dictates PAM specificity, DNA hybridization and Cas activation, followed by site-specific cleavage (Figure 1). Cas9 and Cas12a (Cpf1) are both RNA-guided endonucleases that belong to the Class 2 CRISPR–Cas systems. The Cas9 protein contains HNH and RuvC nuclease domains, while Cas12a harbors RuvC and a putative nuclease (Nuc) domain, as it lacks an HNH domain. Even though each of these nuclease domains can produce single-strand breaks, both domains generate double-strand breaks (DSBs) when expressed simultaneously (13). Eukaryotes mainly repair DSBs through error-prone non-homologous end joining (NHEJ) and by microhomology-mediated end joining (MMEJ) mechanisms that lead to the accumulation of small nucleotide insertions or deletions (indels). Alternatively, DSBs can be repaired by error-free homology-directed repair (HDR) when a template that is homologous to the target site is delivered. However, HDR has a lower efficiency than the error-prone mechanisms, and both approaches have been harnessed for genome editing using catalytically active Cas9 proteins.

The Cas9 protein from *Streptococcus pyogenes* (SpCas9) comprises 1368 amino acids (aa), and it has been catalytically inactivated by mutation of the Asp10 and His840 positions of the HNH and RuvC domains, respectively, to alanine residues (D10A/H840A). The resulting nuclease dead Cas9 (SpdCas9) is the most commonly employed for epigenetic editing. It recognizes the most simple 5′-NGG-3′ PAM sequence, which occurs every 8–12 base pairs in the human genome (11,14–16).

The smaller Cas9 from *Staphylococcus aureus* (SaCas9, comprising 1053 aa) has similarly been catalytically inactivated (D10A/N580A) (SadCas9) for epigenome editing (17). This orthologue variant of dCas9 recognizes a more complex PAM sequence (5′-NNGRRT-3′) (17), and it can be exploited, in conjunction with SpdCas9, for the delivery of multiple epigenetic modifiers to achieve simultaneous gene activation and repression within the same cell (18–21). As summarized in the following sections, its smaller size has been exploited to generate more efficient delivery systems for CRISPR.

Lastly, Cas12a (or Cpf1, comprising 1300 aa) has been characterized more recently and repurposed for genome and epigenome engineering (12). In contrast to Cas9, which utilizes an NGG PAM on the 3′ end of the gRNA, the Cas12a enzyme recognizes a T-rich PAM sequence (5′-TTTV-3′) on the 5′ of the gRNA to cleave the DNA. Catalytically dead Cas12a from *Lachnospiraceae bacterium* (LbdCas12a) has been engineered by (D832A and/or E925A) mutations and it has been successfully adapted for gene transactivation (22,23). The resulting nuclease-null mutants (dCas9, dCas12a) are, therefore, unable to cut the DNA, but they are still able to bind tightly to the nucleic acid, via a programmable gRNA complementary to a specific genomic region, which facilitates different strategies to target EDs for epigenome engineering.

## ENZYMES AND EFFECTOR DOMAINS FOR TRANSCRIPTIONAL ACTIVATION

### Recruiters of endogenous transcriptional activators

**Figure 2.**
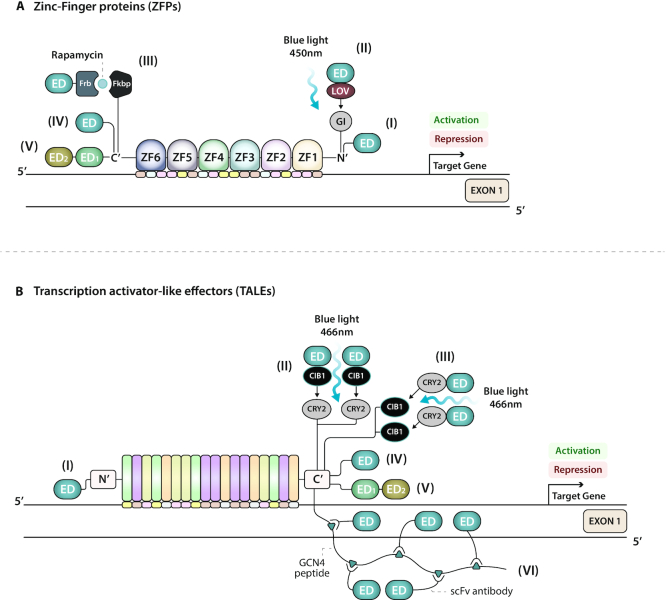
Programmable DNA-targeting platforms for epigenetic editing. (**A**) ZFPs are artificial protein modules that bind to the major groove of DNA. Each zinc finger domain recognizes a 3-nucleotide sequence. Fusions of six ZFPs can recognize an 18-base-pair sequence. (I) A single effector domain (ED) directly fused to the N-terminus of ZFP. (II) A light-inducible system based on blue light that controls heterodimerization of the GIGANTEA (GI) protein fused to ZFP and another plant protein LOV to translocate ED to the gene of interest. (III) A chemically inducible system based on rapamycin. A fusion of ZFP to the human protein Fkbp interacts, in the presence of rapamycin, with a domain derived from human protein Frb linked to ED. This system has also been fused with CRISPR/dCas proteins (see Figure 4A-III). (IV) A single ED directly fused to the C-terminus of ZFP. (V) A bipartite ED system directly linked to the C-terminus of ZFP. (**B**) TALEs are highly conserved tandem repeats or monomers of 34 amino acids in length that only differ in the amino acid residues at the 12th and the 13th position. The amino acids at these two sites in each monomer target a single nucleotide in one DNA strand according to a specific code (NI = adenine, HD = cytosine, NN = guanine, and NG = thymine). Fusions of customizable modules can target an 18-base-pair sequence. (I) A single ED directly fused to the N-terminus of TALE. (II) Optogenetic modulation of gene transcription by the Light-Inducible Transcriptional Effectors (LITE) system. Blue light triggers the interaction between TALE fused to the plant light-sensitive cryptochrome 2 (CRY2) protein and its interacting partner CIB1 linked to ED. (III) A spatiotemporal light-inducible system based on an inverted heterodimerizing fusion protein approach. This system has also been fused with CRISPR/dCas9 (see Figure 4A-I). (IV) A single ED directly fused to the C-terminus of TALE. (V) A bipartite ED system directly linked to the C-terminus of TALE. (VI) The SunTag system C-terminally fused to TALE. This technology involves a protruding GCN4 peptide that contains several antibody-binding sites (triangles) that can recruit multiple single-chain antibodies (scFv) fused to EDs for amplification of epigenetic editing activity. The system has also been devised with CRISPR/dCas proteins (see Figures 3A-V and 4A-IV).

The first epigenome engineering architectures were ZFPs C-terminally fused to activation domains, such as the Virion protein 16, VP16, to recruit the Pol II transcription machinery (24,25). These designs were tailored for reactivation of tumor suppressor genes (TSGs) silenced by epigenetic mechanisms, such as *MASPIN* in breast (26) and ovarian cancer (27). Another transactivator domain, p65 (RelA), has demonstrated potent activation when linked to ZFs (25,28), TALEs (29), SpdCas9 (30), and LbdCas12a for gene multiplex perturbation library screenings (22) (Figures 2A-IV, B-IV, 3A-I).

**Figure 3.**
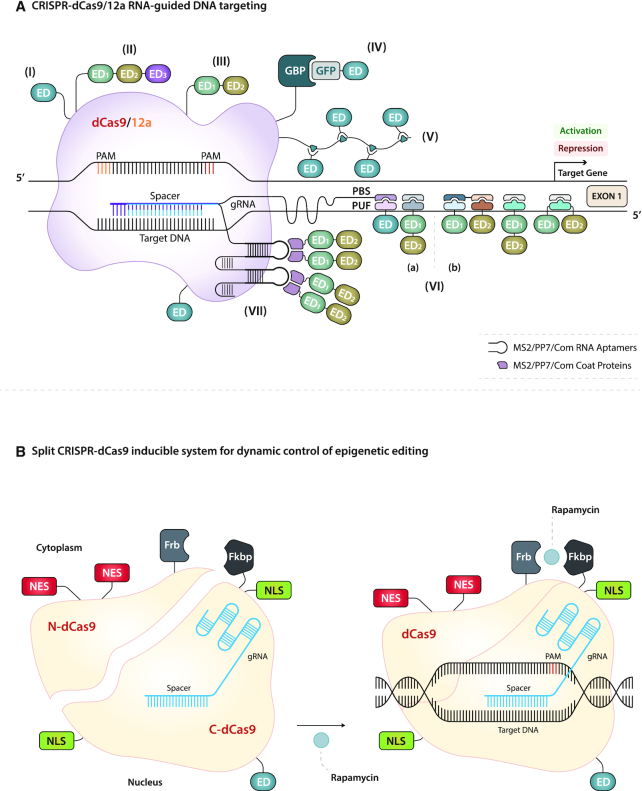
CRISPR-dCas9 and 12a proteins for epigenome engineering. (**A**) dCas9 and 12a are guided to the DNA by a customizable guide RNA (gRNA) and CRISPR RNA (crRNA), respectively. The spacer is the interchangeable portion of the gRNA and crRNA that is complementary to the targeted DNA sequence, which is 20 nucleotides (blue) and 24 nucleotides (violet) in length for dCas9 and dCas12a, respectively. In order to recognize and bind the genomic sequence, dCas proteins also require a protospacer adjacent motif (PAM) immediately 3′ (red) and 5′ (orange) of the target DNA, for dCas9 and dCas12a, respectively. (I) A single ED directly fused to either the N- or the C-terminus of dCas. (II and III) A tripartite and a bipartite ED system directly linked to either the N- or the C-terminus of dCas. (IV) A modular recruitment system based on green fluorescent protein (GFP)-coupled ED via GFP-binding protein (GBP) fused to dCas proteins. (V) The SunTag system fused to dCas proteins for augmentation of epigenetic editing. (VI) The Casilio recruitment platform comprises an appended gRNA fused with one to five copies of Pumilio/FBF (PUF) binding sites (PBS) to recruit multiple distinct EDs, fused to a PUF domain. (a) Simultaneous gene activation and gene repression via EDs, independently recruited, by separate dCas9 proteins targeting different promoters within the same cell. (b) Enhanced gene activation via synergistic activities of distinct EDs recruited, in different combinations, via the same dCas9 protein. (VII) The Synergistic Activation Mediator (SAM) and gRNA 2.0 technology is based on a gRNA modified with MS2, PP7, or Com RNA aptamers from bacteriophages, which recruit EDs fused to aptamer coat proteins to enhance the epigenetic editing activity of dCas proteins already fused to EDs. (**B**) Schematic representation of the split dCas9 strategy for chemical induction of epigenetic editing. The two split fragments, N-dCas9 and C-dCas9 fused to ED, are joined to the rapamycin-binding domains Frb and Fkbp, respectively. Spatial sequestration inside the cell is maintained by an equal ratio of nuclear export sequences (NES) and nuclear localization sequences (NLS) separately fused to the two segments. The addition of rapamycin activates rapid and reversible dCas9 dimerization, thereby allowing dynamic control of transcriptional modulation.

Moreover, transcriptional activation is greatly enhanced when multiple copies (arrays) of transcription activators are assembled (31). For example, synthetic tetrameric repeats of VP16 (VP64) have been linked to ZFs (32), TALEs (9,29,33), and more recently to dCas9 (34–37). Similarly, VP48 and VP160 have also been engineered to a doxycycline-inducible dCas9 system for multiplexed gene activation in mouse embryonic stem cells (ESCs) (38).

Transactivator domains have been engineered for functional screenings in mammalian cells. Despite binding to multiple targets, large libraries of ZFs-VP64 have enabled the identification of malignant gene signatures in head and neck cancer (39,40). In contrast, dCas9-VP64 libraries demonstrated high selectivity and negligible off-target effects, which facilitated the discovery of enhancers associated with human immune dysfunction (41).

Tanenbaum *et al.* developed an epitope-based assembly system (SUperNova Tagging (SunTag)) for effective recruitment of multiple EDs (42). SunTag technology consists of SpdCas9 engineered with a recombinant GCN4 peptide array (10 copies) that is able to recruit multiple nuclear localization signal (NLS)-tagged anti-GCN4 scFv antibodies fused to the EDs. VP64 was the first transactivator investigated in this system, exhibiting more robust gene activation and associated biological responses compared to those achieved by the single gRNA/dCas9-VP64 fusion. The SunTag strategy bypasses the technical challenge of cloning multiple copies of EDs directly onto dCas9 and it circumvents the linker optimization between EDs to achieve high epigenetic activity (Figure 2B-VI, 3A-V). Applications of the SunTag-VP64 system include positive modulation of the *Frataxin* gene in a mouse model of Friedreich ataxia by Platinum TALE (43), dCas9-mediated reactivation of latent HIV-1 in latently infected human T-cell lines (44), the generation of CRISPR activation libraries to identify TSGs and to map complex networks involved in cell differentiation (45), and for genome multiplexing employing LbdCas12a scaffolds (23) (Table 1).

**Table 1.** Epigenetic editing technologies for gene transcriptional activation

Gene regulation: ACTIVATION	Effector domain (ED)	Molecular function	Targeted genomic region	Epigenetic technology (direct ED fusion)	Epigenetic technology (ED recruitment)
**Recruiters of endogenous transcriptional activators**	**VP16** Virion protein 16, from herpes simplex (minimal unit)	DNA demethylation Increased H3K27ac and H3K4me	Gene promoters	ZFP (24,25)	ZFP (GI-LOV) (124)
	**VP48** (3 × VP16)	DNA demethylation Increased H3K27ac and H3K4me	Gene promoters	SpdCas9 (38)	
	**VP64** (tetrameric repeat of VP16)	DNA demethylation Increased H3K27ac and H3K4me	Gene promoters and enhancers	ZFP (26,27,32,40,83) TALE (9,29,33,37) SpdCas9 (30,34–37,41,66,134,254,259) SadCas9 (251)	TALE-SunTag (43) TALE-LITE (120) SpdCas9 split (Fkbp/Frb) (122) SpdCas9 (MS2-MCP) (117) SpdCas9 (MS2-MCP) (DHFR-DD and AID systems) (128) SpdCas9 (LACE) (126) SpdCas9-SunTag (42,44,45) LbdCas12a-SunTag (23)
	**VP160** (10 tandem copies of VP16 motif)	DNA demethylation Increased H3K27ac and H3K4me	Gene promoters	SpdCas9 (38)	
	**VP192** (12 tandem copies of VP16 motif)	DNA demethylation Increased H3K27ac and H3K4me	Gene promoters	SpdCas9 (DHFR-DD system) (127)	
	**p65** (major subunit of NF-kB transcription factor)	Increased H3K9ac and H3K14ac	Gene promoters	ZFP (25,28) TALE (29) SpdCas9 (30)	ZFP (Fkbp/Frb) (121) LbdCas12a (DmrA/DmrC) (22)
	**p65-HSF1** (p65 and Heat shock factor 1)	Increased H3K4me3 and H3K27ac	Gene promoters		SpdCas9 (MS2-MCP) (250)
	**VP64**, **p65-HSF1** Synergistic activation mediator (**SAM**) (Enhanced gene activation)	Not evaluated	Gene promoters		SpdCas9 (MS2-MCP) (46–48)
	**VPR V**P64, **p**65, **R**ta (Enhanced gene activation)	Not evaluated	Gene promoters and enhancers	SpdCas9 (49,51–54,133,235,258) SadCas9 (253) Sp and Sa dCas9 (19) enAsdCas12a (232) PaeCascade (234)	Sp and Nme dCas9 (Fkbp/Frb) and (GAI/GID1) (119) Sp and Sa dCas9 (GAI/GID1) and (ABI/PYL1) (18) LbdCas12a (DmrA/DmrC) (22)
	**VPH V**P64, **p**65, **H**SF1 (Enhanced gene activation)	Not evaluated	Gene promoters		Sp and Sa dCas9-SunTag (4-OHT) (123)
**Enzymes: DNA demethylation**	**TET1** Ten-Eleven Translocation 1 catalytic domain (CD)	Methylcytosine dioxygenase 1 Decreased cytosine methylation (5mC) (eraser)	Gene promoters and enhancers	ZFP and TALE (58) SpdCas9 (60–62,66) SadCas9 (19)	TALE (CRY2-CIB1) (125) SpdCas9 (MS2-MCP) (63) SpdCas9-SunTag (64)
			lncRNA promoters	TALE (59)	
			Major satellite repeats		SpdCas9 (GBP-GFP) (65)
	**TET2** Ten-Eleven Translocation 2 (CD)	Methylcytosine dioxygenase 2 Decreased 5mC (eraser)	Gene promoters	ZFP (55,56)	
	**TDG** Thymine DNA glycosylase	Recognizes and binds 5fC and 5caC and mediates base-excision repair (BER) (eraser)	Gene promoters	ZFP (57)	
	**TET1-CD** and **VP64** (Enhanced gene activation)	DNA Demethylation Increased H3K27ac and H3K4me	Gene promoters		SpdCas9-SunTag (247)
	**TET1-CD** and **GADD45A** or **NEIL2** (Enhanced gene activation)	DNA demethylation coupled with BER machinery (eraser)	Gene promoters		SpdCas9 (Casilio system) (67)
**Multiple distinct EDs for epigenetic memory**	**TET1-CD** and **VPR**	Long-term gene activation DNA demethylation coupled with VPR activator	Gene promoters	Sa and Sp dCas9 (19)	
**Enzymes: Histone lysine acetylation**	**p300** (catalytic domain)	Histone lysine acetyltransferase (KAT) Increased H3K27ac (writer)	Gene promoters and enhancers	ZFP, TALE, and Sp and Nme dCas9 (69) SpdCas9 (70,135) LbdCas12a (23) EcoCascade (233)	
			Putative regulatory elements	SpdCas9 (71) SpdCas9 (AID system) (129)	
	**CBP** (CREB-binding protein domain)	Histone lysine acetyltransferase (KAT) Increased H3K27ac (writer)	Gene promoters and enhancers		SpdCas9 (Casilio system) (68)
	**GCN5** (General Control Of Amino Acid Synthesis Protein 5-Like 2)	Histone lysine acetyltransferase (KAT) Increased H3K9ac and H3K14ac (writer)	Gene promoters	SpdCas9 (72)	
**Chromatin readers**	**BRD4** Bromodomain containing 4	Recognizes and binds acetylated histones, i.e., H4K5ac and H4K8ac (reader)	Gene promoters	SpdCas9 (73)	
**Chromatin remodelers**	**Ldb1** LIM domain binding protein 1 (Self-association domain)	Recruitment of enhancer-associated endogenous Ldb1 Forced looping between promoter and enhancer	Gene promoters and enhancers	ZFP (74) TALE (75)	
**Enzymes: Histone lysine methylation**	**PRDM9** (PR/SET Domain 9)	Histone lysine methyltransferase (KMT) Increased H3K4me3 (writer)	Gene promoters	ZFP and SpdCas9 (76)	
	**DOT1L** (DOT1 Like)	Histone lysine methyltransferase (KMT) Increased H3K79me2 and 3 (writer)	Gene promoters	ZFP and SpdCas9 (76)	
	**MLL3SET** (Myeloid/Lymphoid Or Mixed-Lineage Leukemia Protein 3 SET domain)	Histone lysine methyltransferase (KMT) Increased H3K4me1 (writer) Recruitment of p300 Increased H3K27ac	Super enhancers	SpdCas9 (77)	
	**SMYD3** (SET and MYND Domain Containing 3)	Histone lysine methyltransferase (KMT) Increased H3K4me3 (writer)	Gene promoters	SpdCas9 (78)	
**Multiple distinct EDs for epigenetic memory**	**PRDM9** and **DOT1L**	Long-term gene activation Increased H3K4me3 and H3K79me2 and 3	Gene promoters	SpdCas9 (76)	

**Abbreviations:** H3: histone 3; K: lysine; ac: acetylation; me: methylation; me2: di-methylated state; me3: tri-methylated state; ZFP: zinc-finger proteins; GI: GIGANTEA; LOV: light oxygen voltage domain of FKF1; TALE: transcription activator-like effector; Sp: *Streptococcus pyogenes*; Sa: *Staphylococcus aureus*; dCas9: catalytically deactivated Cas9 protein; SunTag: SUperNova Tagging; LITE: Light-Inducible Transcriptional Effector; FKBP: FK506-binding protein; FRB: FKBP–rapamycin binding; MS2: RNA aptamer; MCP: MS2-coat protein; LACE: Light-Activated CRISPR-dCas9 Effector; Lb: *Lachnospiraceae bacterium*; dCas12a: catalytically deactivated Cas12a protein; DHFR-DD system: dihydrofolate reductase (DHFR)-derived destabilization domain; Rta: replication and transcription activator; en: enhanced; As: *Acidaminococcus sp*.; Pae: *Pseudomonas aeruginosa*; Cascade: CRISPR-associated complex for antiviral defense; DmrA: FKBP domain; DmrC: FRB domain; Nme: *Neisseria meningitidis*; GAI: gibberellin (GA) insensitive protein; GID1: gibberellin-insensitive dwarf1 protein; ABI: abscisic acid (ABA)-insensitive 1 protein; PYL1: abscisic acid receptor; 4-OHT: 4-Hydroxytamoxifen; 5mC: 5-methylcytosine; CRY2: cryptochrome 2; CIB1: cryptochrome-2-interacting binding protein-1; lncRNA: long non-coding RNAs; GFP: green fluorescent protein; GBP: GFP-binding protein; 5fC: 5-formylcytosine; 5caC: 5-carboxylcytosine; GADD45A: Growth Arrest and DNA-Damage-inducible Alpha; NEIL2: Nei-Like DNA Glycosylase 2; Eco: *Escherichia coli*; AID: Auxin-Inducible Degron; H4: histone 4.

The aforementioned approaches rely on the fusion of synthetic DBDs with the same type of EDs, which inevitably leads to depletion of the same endogenous co-factors that are recruited. To mimic the natural context of gene transcription, where endogenous TFs perform synergistically with a variety of co-factors, new strategies aim to combine mechanistically distinct EDs in order to improve activation efficiencies.

By modifying the gRNA scaffold without affecting the DNA binding capacity of dCas9, Konermann *et al.* were able to recruit multiple distinct EDs with a single gRNA (46) (Figure 3A-VII). Two stem-loops of the modified gRNA protrude from the SpdCas9/gRNA/target DNA tertiary complex, and they are extended with MS2 RNA aptamers from MS2 bacteriophage. The EDs, such as p65 and HSF1, are fused to the MS2 coat protein (MCP), which is recognized and recruited by the Synergistic Activation Mediator (SAM) system, thereby giving rise to a synergistic effect in combination with dCas9 linked to VP64. This system can result in robust gene activation and gene reprogramming for potential treatment of male androgen deficiency diseases (47). Moreover, Fidanza *et al.* created the UniSam system, which is an all-in-one vector version of dCas9-SAM technology to maximize post-transfection cell viability by reduction of the total amount of DNA plasmid that is used (48). Both SAM and SunTag technologies have the advantage of being more scalable, in particular for genome-wide gain-of-function screens and for genome multiplexing relative to tiling of gRNAs (30,34–36), while still achieving the same activation efficiency.

George Church's laboratory has C-terminally functionalized SpdCas9 with a tripartite VPR system (49). The VPR is a tandem chimeric fusion of VP64-p65-Rta, the Rta being another transcriptional activator adopted from gammaherpesviruses (50) (Figure 3A-II). The dCas9-VPR construct has demonstrated superior potency than dCas9-VP64 in terms of multi-activation of a panel of genes, thus providing a powerful tool for cellular identity reprogramming, such as the neuronal differentiation of human iPSCs (49), as well as for rescuing disease-causing mutations in genetic disorders, such as cystic fibrosis (51). In oncology, dCas9-VPR has demonstrated pronounced upregulation of TSGs, such as *PTEN* in triple-negative breast cancer and melanoma (52), *DKK* in prostate cancer (53), and *MASPIN* and *REPRIMO* in lung and gastric cancer cells (54).

Importantly, additional research is needed, however, to assess how all of these second-generation activators differ in terms of their molecular mechanisms and synergistic effects when co-recruited at particular (epi)-genomic contexts. To this aim, numerous epigenetic enzymes fused to programmable DBDs have recently been developed for precise control of gene regulation.

### Epigenetic enzymes for gene activation

#### DNA demethylases (erasers)

Aberrant DNA methylation (DNAme), i.e., 5-methylcytosine, is associated with many diseases, such as neoplasms and neural degeneration. A ZFP engineered with the catalytic domain (CD) of TET2, Ten-Eleven Translocation methylcytosine dioxygenase 2 (ZFP-TET2), was first reported to precisely direct demethylation and reactivation of epigenetically silenced TSGs in cervical (55) and ovarian (56) cancer cells. Thymine DNA glycosylase (TDG) is another enzyme implicated in the cascade of methylcytosine demethylation. A quartet of ZFP-TDGs concurrently targeting the *NOS2* promoter induced gene reactivation in fibroblasts (57) (Figure 2A-IV).

To understand the causal effect of DNA demethylation and activation of gene expression, J. Keith Joung's laboratory has engineered TALEs linked to the hydroxylase activity of the human TET1 CD to identify and remove critical CpG dinucleotides methylation marks at endogenous gene promoters in a chronic myelogenous leukemia cell line (58). Similarly, TALE-TET1 and dCas9-TET1 fusions were employed as a way to treat diabetes via induction of β cell replication (59), to reactivate the TSG *BRCA1* and inhibition of cell proliferation (60), to facilitate reprogramming of fibroblasts into myoblasts (61), and to potentially target diseases caused by dysregulated gene expression, such as Fragile X syndrome (62) (Figures 2B-IV, 3A-I). ED recruitment technologies have also been successfully applied to manipulate DNA demethylation with TET1-CD, as a fusion with dCas9 or MS2 coat proteins in neuroblastoma cells, with negligible off-target effects (63). On the other hand, an optimized SunTag strategy with an extended linker length between the GCN4 peptide arrays, from 5 to 22 aa, and a reduction in the number of GCN4 copies, effectively recruited TET1-CD to functionally relevant CpGs sites in ESCs, primary neural precursor cells, cancer cell lines and mouse fetuses (64).

Alternatively, a doxycycline-inducible modular recruiting system based on dCas9 fused to green fluorescent protein (GFP) Binding Protein (GBP) and GFP-TET1-CD represents an additional platform to simultaneously titrate the two molecular constituents, thus minimizing off-target effects (65) (Figure 3A-IV).

When DNAme exerts tight control over gene expression through sustained silencing, as is the case for *SOX1* in neural progenitor cells, a combination of different EDs targeting the same region, such as dCas9-VP64 and dCas9-TET1-CD, is required to remove cell identity barriers, thereby leading to transcriptional reprogramming (66). TET1-CD and VPR, N-terminally fused to SadCas9 and SpdCas9 orthologues, respectively, represent further examples of a potent synergistic effect that has led to *HNF1A* gene expression being maintained for up to 30 days (19) (Figure 3A-I).

Lastly, another strategy to enhance activation of methylation-silenced genes relies on linking TET1-CD activity with the Base Excision Repair machinery, such as Growth Arrest and DNA-Damage-inducible Alpha (GADD45A) or Nei-Like DNA Glycosylase 2 (NEIL2) to complete the DNA demethylation cycle (67). Multimerization of EDs at targeted sites has been accomplished by coupling, in different combinations, distinct EDs to the PUMILIO/FBF (PUF) modules, referred to as the Casilio recruiting technology (68). This consists of SpdCas9 protein and an appended gRNA with one to five copies of PUF Binding Site (PBS), while the EDs are fused to PUF modules (Figure 3A-VI-b).

#### Histone lysine acetyltransferases (KATs) (writers)

Hilton *et al.* have shown that dCas9 fused to the CD of the human acetyltransferase p300, which catalyzes H3K27 acetylation in promoters and in proximal and distal enhancers, induced gene expression with a high degree of specificity across the genome (69). Applications of SpdCas9-p300 range from activation of immune response regulation (70) to the discovery of new regulatory elements (71). Lastly, epigenetic editing in cancer cells has also been achieved with LbdCas12a-p300 (23) (Figure 3A-I).

In another study, the histone lysine acetyltransferase (KAT) domain from CREB-binding protein (CBP) was C-terminally engineered with dCas9 as well as with the Casilio PUF modules (68). The recruited EDs exhibited a higher efficacy in regulating gene expression by targeting both proximal and distal enhancers compared to the direct dCas9-ED fusions (Figure 3A-I, VI-a).

Lastly, the C-terminal domain of dCas9 has also been engineered with the enzymatic core of KAT GCN5 from *P. falciparum* to hyper-acetylate the transcriptional start site of the silent *Rh4* gene, as a way to block the invasion of human erythrocytes by malaria parasites (72) (Figure 3A-I).

#### Chromatin readers

Catalytically deficient dCas9 has been fused with BRD4, a bromodomain reader of acetyl-lysine histones, to reinforce the effect of epidrugs for the treatment of neuropsychiatric disorders. The engineering of dCas9-BRD4 resulted in enhanced *BDNF* gene expression, neuroplasticity, and memory following pharmacological inhibition of HDAC3 (73) (Figure 3A-I).

#### Chromatin remodelers

Overruling chromatin conformations that instruct and restrict gene expression programs, such as the synthesis of γ-fetal and β-adult globins, can be achieved by forcing chromatin looping. To this aim, ZFPs (74) and TALEs (75) fused to the enhancer recruiter, LIM domain-binding protein 1 (Ldb1), targeting the developmentally silenced embryonic globin gene, were able to induce its *de novo* transcriptional activity in adult murine erythroblasts and in human umbilical cord blood-derived erythroid progenitor cells, respectively (Figure 2A-IV, B-IV).

#### Histone lysine methyltransferases (KMTs) (writers)

Marianne Rots’ laboratory first demonstrated that targeted deposition of H3K4me3 by fusion of the catalytic core domain of the histone-lysine *N*-methyltransferase PR/SET Domain 9 (PRDM9) to either dCas9 or ZFPs is sufficient to re-express silent genes in a context-dependent manner in lung, ovarian and cervical cancer cells. However, H3K79me induced by dCas9 or ZFPs fused to the DOT1-like histone-lysine *N*-methyltransferase, H3 lysine-79 specific (DOT1L) was found to be essential to maintain durable gene re-expression (76) (Figures 2A-IV and 3A-I).

The myeloid/lymphoid or mixed-lineage leukemia protein 3 SET domain (MLL3SET) is another histone lysine methyltransferase (KMT) that was recently linked to dCas9 to orchestrate chromatin interactions between super-enhancers and the *SOX2* promoter in differentiating mouse ESCs (77). Furthermore, an N-terminally truncated variant of the KMT SMYD3 that is incapable of binding to its DNA cognate motif 5′-CCCTCC-3′ nor able to interact with the endogenous positive coactivator 4, PC4, has been tethered to dCas9 to boost gene transcription, thereby expanding the arsenal of tools available for epigenome editing (78) (Figure 3A-I).

In summary, upregulation of silenced genes, particularly via ‘hit-and-run’ approaches (transient transfections) by single EDs, either directly fused to or recruited by DBDs, has been shown to be effective for many target genes. However, multiple and mechanistically distinct EDs are required to avoid exhaustion of the recruited endogenous co-factors, as well as to obtain a more durable epigenome manipulation. This can be achieved by different recruiting systems such as SunTag, aptameric, and Casilio technologies. There are several limitations, however, for each system, which include the large size of the SunTag constructs, which can affect delivery efficiencies *in vivo*, and the number of available aptameric sequences that can be incorporated in SAM systems (Table 2).

**Table 2.** Comparison of different assembly methodologies of effector domains (EDs) for epigenome engineering

Strategies	Advantages	Disadvantages
**Single ED** directly fused to DBDs	- Small size potentially facilitates delivery in target cells and tissues - Cost-effective to produce, e.g., by recombinant protein production	- Limited epigenetic editing ability associated to single EDs - Requires *de novo* construction of each DBD - Single EDs may exhaust endogenous transcriptional machinery
**Multiple EDs** directly fused to DBDs	- Simultaneous editing of multiple epigenetic marks to restore long-lasting manipulation of the epigenome or ‘epigenetic memory’ - Versatile/flexible enabling N- and/or C-terminal fusions of arrays of effectors - Delivery is facilitated by ‘all-in-one’ component system	- Larger size of ED arrays could affect intracellular delivery and potentially change the specificity - ED combinations may have to be tailored to genomic contexts
**SunTag system**	- Enhances epigenetic editing activity - Low frequency of off-target effects due to fewer DBDs employed nor gRNA tiling	- Larger size of the multi-component systems limits intracellular delivery - Requires linker optimization of the GCN4 polypeptide array
**GBP-GFP system**	- Any ED can be potentially GFP-tagged - Fluorescent tagging enables dynamic real-time microscopy visualization of dCas9	- GFP fusions may potentially interfere with epigenetic editing activity
**Aptameric systems**	- Enhances epigenetic editing activity - Enables targeting of multiple EDs - Low frequency of off-target effects, nor gRNA tiling - Enables simultaneous gene activation and repression	- Delivery limited by a three-component system - Potentially limited by the number of available RNA aptamers (MS2, PP7, Com) - Requires delivery of larger gRNAs and co-delivery of multiple coat proteins
**Casilio system**	- Highly flexible ED recruiting module design - Highly controlled stoichiometry of EDs - Potential for multiplexing and multimerization of synergistic EDs - Enables robust epigenetic editing activity and longevity - Facilitates simultaneous gene activation and gene repression	- Delivery limited by a three-component system

**Abbreviations:** ED: effector domain; DBD: DNA-Binding Domain; SunTag: SUperNova Tagging; GFP: green fluorescent protein; GBP: GFP-binding protein.

## ENZYMES AND EFFECTOR DOMAINS FOR TRANSCRIPTIONAL REPRESSION

Targeted gene repression has been applied in basic and translational research, ranging from loss-of-function screening with minimal off-target activity (45) to a more precise strategy to switch-off oncogenic addictions with long-lasting effects, such as *SOX2* in breast cancer (79–81).

### Recruiters of endogenous transcriptional repressors

The repressor domain most commonly fused to DBDs is the Krüppel-associated box KRAB (82). ZF arrays have been N-terminally tethered to KRAB to down-regulate genes (83), laying the foundations of synthetic biology for gene therapy applications (79). Similarly, TALE-KRAB fusions allowed potent, specific, and simultaneous gene knock-downs to investigate interconnected molecular pathways (84). Moreover, the KRAB domain fused to either the N- or the C-terminus of dCas9 has been devised in a doxycycline-inducible system for robust targeted inhibition of gene transcription at promoters (30,45) as well as at enhancers (85). A combinatorial gRNA-dCas9-KRAB system demonstrated epigenetic perturbation of enhancers and super-enhancers. In this approach (Mosaic-seq), the activity of single and multiple gRNAs was monitored at single-cell resolution by screening of a barcode gRNA library followed by sequencing and identification of the gRNA barcodes (86) (Figures 2A-I, B-IV, 3A-I). In addition to KRAB, the SIN3A (87), FOG1 (88) and HP1 (89) are additional examples of EDs recruiting the endogenous repressor machinery, thereby resulting in targeted gene down-regulation (Table 3).

**Table 3.** Epigenetic editing technologies for gene transcriptional repression

Gene regulation: REPRESSION	Effector domain (ED)	Molecular function	Targeted genomic region	Epigenetic technology (direct ED fusion)	Epigenetic technology (ED recruitment)
**Recruiters of endogenous transcriptional repressors**	**KRAB** (Krüppel-associated box)	Recruitment of corepressor KAP1, HP1, SetDB1 and NuRD Increased H3K9me3 Decreased H3K9ac	Gene promoters and enhancers	ZFP (8,79,83,154,191,244) TALE (84) SpdCas9 (30,45,85,86,174) SadCas9 (245) LmoCascade (233)	Sp and Sa dCas9 (GAI/GID1) and (ABI/PYL1) (18) SpdCas9 (Com) (117)
	**SIN3A** (SIN3 Transcription Regulator Family Member A)	Transcriptional corepressor hub Recruitment of HDACs Decreased H3ac	Gene promoters and enhancers	SpdCas9 (87)	TALE-LITE (120)
	**FOG1** (Friend of GATA1)	Recruitment of NuRD, HDAC1 and 2 and PRC2 complex Increased H3K27me3	Gene promoters	SpdCas9 (88)	
	**HP1** (Heterochromatin protein 1)	Interacts with KMTs, e.g., Suv39h1, SetDB1, and G9a Increased H3K9me3	Gene promoters		SpdCas9-FIRE (89)
	**KRAB** and **MeCP2** (Enhanced gene repression)	MeCP2 binds to DNA methyltransferase DNMT1 and the SIN3A–histone deacetylase co-repressor complex	Gene promoters	SpdCas9 (90)	
**Enzymes: DNA methylation (DNAme)**	**DNMT3A** (catalytic domain or full-length)	DNA methyltransferase Increased cytosine methylation (writer)	Gene promoters and introns	ZFP (80,81,92,243,246) SpdCas9 (61,93–97,105) SadCas9 (20)	TALE (CRY2-CIB1) (125) SpdCas9-SunTag (106,107)
			Major satellite repeats		SpdCas9 (GBP-GFP) (65)
	**DNMT3B** (catalytic domain)	DNA methyltransferase Increased cytosine methylation (writer)	Gene promoters	SpdCas9 (105)	
	**DNMT3A** and **DNMT3L** (Enhanced gene repression)	A: DNA methyltransferase (writer) L: regulatory factor	Gene promoters	ZFP (100) TALE (103) SpdCas9 (101,102)	
	**MQ1**	Prokaryotic DNA methyltransferase (writer)	Gene promoters	SpdCas9 (98)	
	**M.SssI**	Prokaryotic DNA methyltransferase (writer)	Pericentromeres	TALE and SpdCas9 (104)	
**Multiple distinct EDs for epigenetic memory**	**DNMT3A**, **DNMT3L**, and **KRAB**	Long-term gene silencing Increased DNAme and H3K9me3 Decreased H3K4me3	Gene promoters	TALE (108,109) SpdCas9 (108)	
**Enzymes: Histone lysine methylation**	**Ezh2** (catalytic domain)	Histone lysine methyltransferase (KMT) Increased H3K27me3 (writer)	Gene promoters	SpdCas9 (88,110,112)	SpdCas9 (PP7-PCP) (111)
	**G9a** (EHMT2) (minimal catalytic domain)	Histone lysine methyltransferase (KMT) Increased H3K9me2 (writer)	Gene promoters	ZFP (28)	
**Multiple distinct EDs for epigenetic memory**	**Ezh2**, **DNMT3A**, and **DNMT3L** (overexpressed)	Long-term gene silencing Increased DNAme and H3K27me3 Decreased H3K27ac	Gene promoters	SpdCas9 (112) (Ezh2 and DNMT3A)	
**Enzymes: Histone lysine demethylation**	**LSD1** (Lysine Demethylase 1A)	Histone lysine demethylase (KDM) Decreased H3K4me2 Decreased H3K27ac (eraser)	Enhancers	TALE (113) NmedCas9 (114) SadCas9 (21)	
**Enzymes: Histone lysine deacetylation**	**HDAC3** (full-length)	Histone deacetylase Decreased H3K27ac (eraser)	Gene promoters	SpdCas9 (115)	
	**HDAC8** (full-length)	Histone deacetylase Decreased H3K27ac (eraser)	Enhancers	SpdCas9 (116)	
	**Sir2a** (Sirtuin Type 1)	Histone deacetylase Decreased H3ac and H4ac (eraser)	Gene promoters	SpdCas9 (72)	

**Abbreviations:** KAP1: KRAB associated protein 1; HP1: heterochromatin protein 1; SetDB1: SET domain bifurcated histone lysine methyltransferase 1; NuRD: nucleosome remodeling and deacetylase; KMT: histone lysine methyltransferase; HDAC: histone lysine deacetylase; H3: histone 3; K: lysine; me2: di-methylated state; me3: tri-methylated state; ac: acetylation; ZFP: zinc-finger proteins; TALE: transcription activator-like effector; Sp: *Streptococcus pyogenes*; Sa: *Staphylococcus aureus*; dCas9: catalytically deactivated Cas9 protein; Lmo: *Listeria monocytogenes*; Cascade: CRISPR-associated complex for antiviral defense; GAI: gibberellin (GA) insensitive protein; GID1: gibberellin-insensitive dwarf1 protein; ABI: abscisic acid (ABA)-insensitive 1 protein; PYL1: abscisic acid receptor; Com: RNA aptamer Com-coat protein; LITE: light-inducible transcriptional effector; PRC2: polycomb repressive complex 2; Suv39h1: suppressor of variegation 3–9 homolog 1; G9a: histone-lysine *N*-methyltransferase EHMT2; EHMT2: euchromatic histone lysine methyltransferase 2; FIRE: Fkbp/Frb inducible recruitment for epigenome; FKBP: FK506-binding protein; FRB: FKBP–rapamycin binding; MeCP2: methyl-CpG binding protein 2; DNMT: DNA methyltransferase; CRY2: cryptochrome 2; CIB1: cryptochrome-2-interacting binding protein-1; SunTag: SUperNova tagging; GFP: green fluorescent protein; GBP: GFP-binding protein; Ezh2: enhancer of zeste 2 polycomb repressive complex 2 subunit; PP7: RNA aptamer; PCP: PP7-coat protein; Nme: *Neisseria meningitidis*.

Another approach focuses on the assembly of different ED recruiters for enhanced repression of multiple genes simultaneously (90). A new artificial repressor tool has been engineered by fusion of dCas9 to KRAB and with the methyl-CpG binding protein 2 (MeCP2), thereby generating the bipartite entity dCas9-KRAB-MeCP2 that has been shown to have a superior degree of gene repression than dCas9-KRAB (Figure 3A-III). However, none of these recruiters were able to induce a long-lasting repressive chromatin state, and restoration of gene repression was observed within 5–10 days after the DBD was no longer expressed (45,88,89).

An additional study demonstrated that sustained expression of KRAB for 10 days achieved long-term epigenetic silencing in targeted loci in the context of a doxycycline-inducible articular chromosome, with a resulting memory of ∼60 days (and ∼66% silenced cells in the population). Conversely, *de novo* DNAme induced by DNMT3B led to a similar irreversible gene silencing rate (∼58%) after 3 days of activity (91). Similar findings have been shown in the context of endogenous chromatin in cancer cells *in vivo*, as in the case for ZF-KRAB targeting the *SOX2* gene (79) and ZF-DNMT3A targeting the *MASPIN* (80) and *SOX2* genes (80,81), thereby corroborating the longevity of epigenetic silencing via *de novo* DNAme and consequent inhibition of tumor growth.

### Epigenetic enzymes for gene repression

#### DNA methyltransferases (DNMTs) (writers)

Epigenetic engineering, in particular with DNA synthetic methyltransferases, has led to the development of molecular tools to advance the field of functional epigenetics, notably to understand the causal effects of DNAme in chromatin structure, gene expression, and phenotype specification at particular loci. For example, ZFPs fused to the CD of DNMT3A mediated stable repression of the *SOX2* oncogene *in vivo* (81), and similar designs inactivated the TSG *P16* (92) by writing *de novo* DNAme at specific regulatory regions (Figures 2A-IV and 3A-I). Moreover, site-specific deposition of DNAme mediated by dCas9-DNMT3A enabled investigation of the physiological mechanistic links between *de novo* DNAme and cellular differentiation (93). Furthermore, this approach confirmed that *de novo* promoter hyper-methylation of the TSG *SMARCA2* is an epigenetic driver event in lung adenocarcinoma progression (94). In addition to applications for cancer treatment, synthetic *de novo* DNAme to down-regulate the abnormally high levels of the *SNCA* gene could represent a promising potential treatment for Parkinson's disease (95).

Other studies have revealed the spatiotemporal dynamics of *de novo* DNAme and gene silencing. Importantly, multiple gRNAs are required to synergistically methylate a broader chromosomal region within CpG-islands controlling promoter gene expression (96). In the case of the TSG *CDKN2A*, broad DNAme deposition across the entire CpG-island was required to repress gene expression (97). In regard to the dynamic control of DNAme, it reached its peak of efficacy 3 days (97) and 6–7 days after transfection (96). In another study, Lei *et al.* achieved a DNAme peak in less time, 24 h post-transfection, by directly fusing dCas9 with the prokaryotic DNMT mutant version, MQ1^Q147L^. This approach is particularly important for modeling and to study embryogenesis *in vivo*, where editing of DNAme has to be rapid (98) (Figure 3A-I).

Several strategies based on different assembly methods of diverse EDs have also been devised to increase DNAme activity while minimizing global off-target effects. In terms of DNAme activity, it is known that the catalytic activity of DNMT3A is stimulated by its regulatory cofactor DNMT3L (99). When ZFPs were C-terminally linked to DNMT3A and DNMT3L the resulting constructs yielded 2-fold more gene silencing than DNMT3A alone (100) (Figure 2A-V). Stepper *et al.* have also confirmed the potency of DNMT3A and DNMT3L multimerization attached to dCas9, although unintended off-target effects occurred (101). The dCas9-DNMT3A-DNMT3L fusion has been used to validate the DNAme-mediated silencing of the TSG *CDKN2A* during tumorigenesis (102). Indeed, *CDKN2A* silencing by the TALE-DNMT3A-DNMT3L fusion demonstrated increased cell replication in primary human fibroblasts (103) (Figures 2B-V, 3A-III). In contrast to mammalian methyltransferases, the bacterial DNMT SssI does not require the DNMT3L co-factor. Yamazaki *et al.* have fused SssI to either TALEs or dCas9 to investigate the impact of DNAme on mitotic chromosomal segregation. However, refinements on dCas9-SssI system are required to reduce off-target activity (104).

To investigate the specificity of the SpdCas9-DNMT3A or -DNMT3B, Lin *et al.* conducted whole-genome bisulfite sequencing and they identified off-target differentially methylated regions (DMR)s (105). To reduce off-target DMRs, as well as to extend the DNAme deposition over larger genomic regions (4.5-kb), Huang and colleagues employed a SpdCas9-SunTag-DNMT3A system (Figure 3A-V). This modular platform greatly amplifies the DNMT3A enzyme concentration at the site of interest. This allows the formation of functional DNMT3A tetramers while using a single gRNA, thereby exerting a minimal impact on global DNAme (106). A similar strategy has been exploited followed by a more comprehensive survey of on- and off-targets, revealing the lowest level of DNAme off-targets documented to date, compared to direct fusions (107).

Finally, to achieve long-lasting effects, combinations of distinct EDs, such as KRAB, DNMT3A, and DNMT3L have been separately fused to multiple SpdCas9s or TALEs (108) or linked together as an N-KRAB-TALE-DNMT3A-Dnmt3L-C fusion (109). Both studies have demonstrated local and persistent alteration of the chromatin structure, thus ensuring long-term epigenetic memory in genes involved in immune responses, such as *B2M* (108) or *CCR5* and *CXCR4* in human primary T lymphocytes as a form of protection from HIV infection (109), with negligible off-target activity (Figure 2B-I, V and 3A-I).

#### Histone lysine methyltransferases (KMTs) (writers)

In addition to KRAB and DNMTs, additional enzymes have been harnessed to more readily achieve synthetic and enduring gene silencing. ZF domains engineered with the CD of G9a have been utilized for *in vivo* targeting of the *FosB* gene to elucidate transcriptional gene dysregulation in neuropsychiatric diseases, such as drug-addiction and depression (28) (Figure 2A-IV).

Furthermore, the KMT EZH2 has been attached to dCas9 to down-regulate the *GRANULIN* gene, which is responsible for cancer progression in the hepatoma cell line Hep3B (110). In another study, EZH2 was fused to the PP7 coat protein (PCP) and recruited by the PP7 RNA aptameric system, demonstrating that targeted manipulation of H3K27me3 was inherited by the daughter cells (111) (Figure 3A-I, VII). O’Geen *et al.* co-targeted Ezh2-dCas9 with DNMT3A-dCas9 along with simultaneous overexpression of DNMT3L. This multivalent design induced an epigenetic switch from euchromatin to heterochromatin capable of inducing long-term repression of *HER2* for at least 50 days and perpetuated for 57 cell divisions in the human colon cancer cell line HCT116, with no detectable off-target activity (112). This is another example whereby the combination of multiple distinct EDs was found to be critical for sustained epigenetic modulation (Figure 3A-I).

#### Histone lysine demethylases (KDMs) (erasers)

Targeted decommissioning of endogenous enhancers by TALEs (113) and NmedCas9 from *Neisseria meningitidis* (114) fused to LSD1 has been extremely helpful to researchers. Precise inactivation of the enhancer's chromatin is a powerful tool to functionally characterize these tissue-specific elements for transcriptional repression activity. To this aim, SadCas9-LSD1 has been used to modify the chromatin state of a conserved enhancer, leading to altered expression of PDX1 and its target genes in insulinoma cells and in pancreatic islets (21) (Figure 2B-IV, 3A-I).

#### Histone deacetylases (HDACs) (erasers)

Another new synthetic epigenome remodeler is based on the fusion of SpdCas9 to HDAC3. However, in this study only modest gene repression was observed, suggesting that the chromatin environment plays an important role in gene modulation, particularly when an enzyme that has the ability to bidirectionally control gene transcription, is used to target gene promoters (115). Conversely, SpdCas9-HDAC8 was able to deacetylate enhancers and reduce *Fos* gene expression in neurons (116). In another study, SpdCas9 was fused to Sir2a, an HDAC from *P. falciparum*, to mediate hypoacetylation of the *eba-175* gene, which is the most highly expressed erythrocyte invasion-related gene in *P. falciparum* (72) (Figure 3A-I).

In conclusion, to induce potent and persistent gene silencing, multiple strategies take advantage of the linkage of mechanistically distinct EDs. While DNMTs alone have demonstrated a modest degree of silencing activity, the presence of additional cofactors enables the reinforcement and maintenance of DNAme over cell generations. Additionally, the incorporation of diverse epigenetic effectors, such as KMTs and HDACs, may be required to maximize gene silencing. The specific combinations of EDs harnessed for gene silencing generally depend on the particular epigenomic context targeted; thus, new emerging strategies aim to expand our combinatorial arsenal of epigenetic domains for effective epigenome manipulation.

## SIMULTANEOUS STRATEGIES FOR GENE ACTIVATION AND REPRESSION

Eukaryotic cells execute complex transcriptional programs, for example during development or during metabolic pathway regulation, where specific sets of genes are activated while others are simultaneously repressed. Zalatan *et al.* engineered three separate scaffolding modules fused with the gRNA of SpdCas9, based on the viral RNA sequences of the MS2, PP7 and Com aptamers. These aptamers recognize homodimeric MCP, PCP and Com RNA-binding coat proteins, respectively. The EDs, such as VP64 and KRAB, are then C-terminally fused to different RNA-binding coat protein sequences and recruited by a single gRNA/SpdCas9, thereby enabling concomitant synthetic ON/OFF gene regulation in human cells (117) (Figure 3A-VII). There are, however, limitations to this approach due to the restricted number of well-characterized RNA aptamers as well as potential difficulties in gRNA expression when more copies of these structured aptamers are engineered onto the gRNA. The Casilio technology has been developed to bypass these aforementioned limitations, achieving both multiplexing and multimerization of EDs. A recent publication demonstrated simultaneous activation via p65-HSF1 and repression via KRAB, when the Casilio modules were independently recruited by dCas9 targeting the *OCT4* and *SOX2* gene promoters, respectively. This system has the capability to exponentially increase the number of distinct EDs, including KAT enzymes, to target specific gene networks (68) (Figure 3A-VI-a).

**Figure 4.**
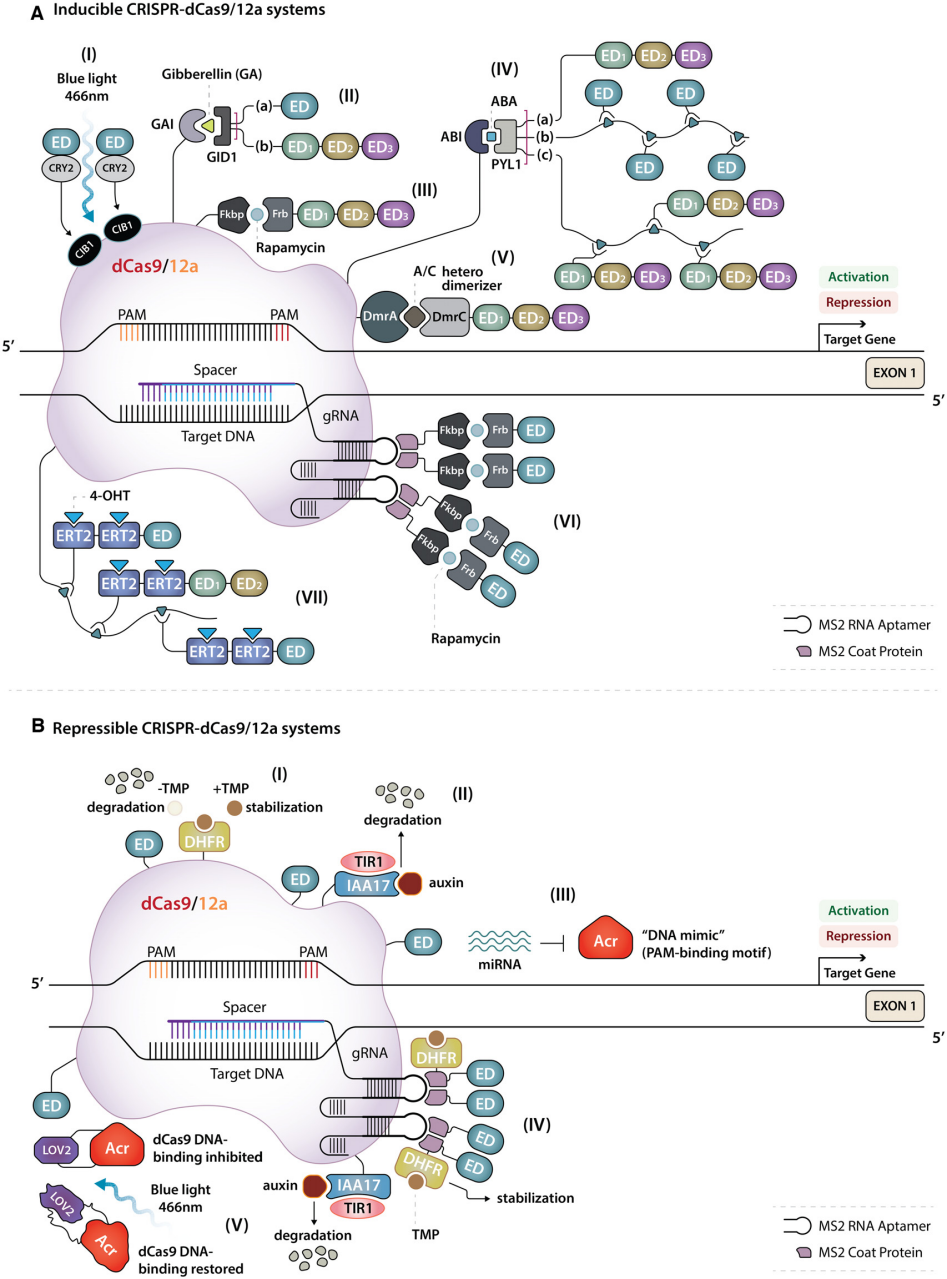
Inducible and repressible systems for precise and dynamic control of CRISPR-dCas9- and 12a-mediated epigenetic editing. (**A**) (I) The light-activated CRISPR-dCas9 effector (LACE) system induces spatiotemporal gene regulation based on exposure to blue light. (II) The chemical gibberellin (GA) induces dimerization of dCas fused to the GA-insensitive (GAI) plant protein and its binding partner gibberellin-insensitive dwarf1 (GID1) linked to either (a) single EDs or (b) tripartite ED systems. (III) The Fkbp-Frb technology recruits tripartite ED systems in the presence of rapamycin. (IV) The chemical abscisic acid (ABA)-inducible system. ABA triggers dimerization of dCas fused to the ABA-insensitive 1 (ABI) plant protein and its PYL1 interacting domain directly linked to (a) tripartite ED systems or to the SunTag system, which recruits (b) single EDs or (c) tripartite ED systems. (V) A drug-dependent system based on DmrA and DmrC domains fused to dCas and EDs, respectively, which interact only in the presence of the rapamycin analog A/C heterodimerizer. (VI) The Fkbp/Frb inducible recruitment for epigenome editing (FIRE) system combines the gRNA MS2 technology with the rapamycin-dependent dimerization approach. (VII) The hybrid drug inducible technology (HIT) based on the SunTag system. scFv antibodies are engineered with two copies of a mutated human estrogen receptor (ERT2) followed by single and multiple EDs. 4-Hydroxytamoxifen (4-OHT) induces nuclear translocation of these constructs that are otherwise retained in the cytoplasm. (**B**) (I) A drug-tunable system for conditional stabilization of dCas linked to ED (dCas9-ED) and N-terminally fused to a dihydrofolate reductase (DHFR)-derived destabilization domain. The addition of trimethoprim (TMP) temporally stabilizes the fusion construct. (II) Auxin-Inducible Degron technology (AID) involves tagging dCas-ED with the auxin plant hormone-sensitive domain IAA17 and co-expression of the auxiliary protein TIR1. The addition of auxin targets the chimeric dCas-ED protein for rapid proteasomal degradation. (III) Anti-CRISPR (Acr) proteins, e.g., AcrIIA4, interfere, and compete with dCas9-ED DNA recognition. The microRNA (miRNA)-responsive ‘Acr switch’ system is designed to cell-specifically regulate epigenetic editing. High levels of a specific miRNA can block Acr expression, thereby releasing dCas9-ED. (IV) A combinatorial strategy that couples the DHFR-TMP system, based on the MS2 ED-recruitment approach, with the AID technology. TMP results in stability, which can be abrogated by the addition of auxin to the system. (V) Optogenetic control based on the CASANOVA system. Acr fused to the plant photosensor LOV2 constitutively interferes with dCas9-ED DNA targeting. Blue light unfolds and impairs Acr-LOV2 fusion, thereby releasing dCas9-ED.

Another strategy deploys different dCas9 orthologues in a dual orthogonal inducible system (18) (Figure 4A-II, IV). An example is the expression of DNMT3A-SpdCas9 and TET1-SadCas9 fusions within the same cells (19). A potential limitation of this approach is the limited frequency of some of the dCas9 orthologues binding to specific promoters relative to that of SpdCas9.

## INDUCIBLE SYSTEMS FOR DYNAMIC CONTROL OF EPIGENETIC EDITING

Novel inducible systems have recently been developed and optimized to finely modulate the expression of epigenetic editors. To this aim, two main strategies have been developed based on chemical and optical systems. Both strategies are based on the principle of oligomerization, in particular, homo- and hetero-dimerization, which mechanistically control the specific activation of proteins and cellular signaling transduction pathways.

For a number of years, orally bioavailable small-molecule drugs, such as doxycycline or tetracycline, have been exploited to induce transgene expression in mammalian cells and mouse models. However, leaky basal gene expression in the absence of the inducers has been noted even after concerted optimization efforts with these expression systems (118). Another concern is the lack of scalability for simultaneous genome multiplexed modulation, as these dimerizer-regulated systems depend on only one inducer. Therefore, other technologies have been developed for tighter epigenetic editing inducibility to assess epigenetic memory (89), to temporally manipulate complex gene networks (18,119), and to establish causal relationships between epigenetic marks, gene regulation, and cellular functions (22,120). Such alternative chemical dimerizers for inducible gene expression *in vitro* and *in vivo* require FDA approval to warrant sufficient safety in terms of bioavailability, biodistribution, drug metabolism, and toxicity. These compounds include rapamycin and its analogs, or rapalogs, such as A/C heterodimerizer; estrogen antagonists, such as 4-hydroxytamoxifen (4-OHT); lastly, plant hormones, represented by the classical antagonistic duo comprising the abscisic acid (ABA)-inducible ABI–PYL1 system and the gibberellin (GA)-inducible GID1–GAI system, for which one of the key features is the tightness of regulation (Figure 4A).

A pioneering study has shown therapeutic gene expression of human growth hormone hGH in a humanized mouse using a tripartite complex comprised of targeted ZF fused to three copies of the cellular protein Fkbp, Frb fused to the transactivator domain p65, and rapamycin acting as an adaptor to join ZF-Fkbp to Frb-p65, thereby resulting in induction of *hGH* gene activation *in vivo* (121) (Figure 2A-III).

Fkbp/Frb Inducible Recruitment for Epigenome editing by the dCas9 (FIRE) system (89) is a recent SpdCas9-MS2 recruitment-based approach that exploits the same dimerizing fusion proteins to reprogram chromatin states in a controllable manner. Recruitment of the endogenous heterochromatin complex comprising methyltransferases such as Suv39h1, SetDB1, and G9a via HP1 locus-specifically deposited H3K9me3 epigenetic mark at the *CXCR4* promoter in HEK293 cells. The gene silencing could be reversed upon washout with FK506, which is a dimeric competitive inhibitor of the dimerizing drug rapamycin (89) (Figure 4A-VI).

With regards to ligand-dependent gene activation, the orthologues SpdCas9 and NmedCas9 were engineered with the chemically induced dimerizing GAI/GID1 and Fkbp/Frb systems, respectively, and fused to VPR to exert simultaneous multiple gene activation with minimal background activity (119) (Figure 4A-II, III).

Synergistic and combinatorial gene activation has also been achieved with LbdCas12a, by leveraging its ability to encode two or more crRNAs in a multiplex single transcript. This drug-dependent system used DmrA and DmrC–VPR fusions in the presence of the rapalog A/C heterodimerizer for controllable multiple gene regulation in the bone osteosarcoma cell line U2OS (22) (Figure 4A-V).

Furthermore, rapid and reversible response to drug induction in transcription modulation has been demonstrated with the split SpdCas9-VP64 system. Spatial sequestration of the two split fragments inside the cells was maintained by an equal ratio of nuclear export sequences (NES) and nuclear localization sequences (NLS) fused to N-dCas9-Frb and C-Fkbp-dCas9-VP64, respectively, and rapamycin was used to activate the dimerization (122) (Figure 3B).

Different dimerizer-inducible systems can be exploited to generate synthetic epigenetic machinery by fusion of distinct EDs to the dCas9 split. Alternatively, different orthologues, such as Sp and SadCas9 fused to VPR and KRAB, and vice versa, can be employed to achieve simultaneous regulation of orthogonal genes when dynamically controlled by ABA- and GA-inducible systems in the same cell (18) (Figure 4A-II, IV).

Lastly, to avoid the use of rapamycin, which interferes with mTOR, a crucial cellular pathway component, Hybrid drug Inducible CRISPR/Cas9 Technologies (HIT) have been developed for rapid and reversible modulation of transcriptional activation of the *OCT4* and *KLF4* genes. The HIT system deploys 4-OHT to chemically induce proximity of either Sp or SadCas9 and the scFv portion of the SunTag molecular entity directly grafted to two copies of a triple-mutated human estrogen receptor (ERT2). ERT2 guarantees low background activity due to its selective affinity for synthetic 4-OHT relative to the endogenous ligand β-estradiol. Without its ligand, scFv-ERT2 is sequestered by heat shock protein 90 in the cytoplasm. VP64 and p65-HSF1 (VPH) are separately attached to scFv-ERT2 constructs, as they have been shown to exhibit more pronounced synergistic activation than V and PH alone or fused together (123) (Figure 4A-VII).

Spatial and temporally tunable gene expression can also be achieved by adopting photoactivatable systems. Initial work combined ZFs fused to GIGANTEA (GI) and VP16 with LOV, two light-inducible dimerizing proteins from *Arabidopsis thaliana*, to control the level of gene activation by modulation of the blue light intensity (124) (Figure 2A-II). Light-inducible transcriptional effector (LITE) systems integrating TALEs fused to the light-sensitive protein CRY2 with its interacting partner CIB1, from *Arabidopsis thaliana*, linked to either VP64 or SIN3 have been used for rapid and reversible optogenetic control of gene activation and repression, respectively, in primary mouse neurons (120). Moreover, exchanging the dimerization domain pair, by fusing TALEs to CIB1 and CRY2 to either DNMT3A-CD or TET1-CD, allowed optical regulation of the methylation state of *Ascl1*, which is a candidate proneuronal gene in murine neural stem cells (125) (Figure 2B-II, III).

Dynamic gene regulation has also been achieved with the light-activated CRISPR–Cas9 effector (LACE) system. The same optogenetic actuators, CRY2 and CIB1, were attached to VP64 and to SpdCas9, respectively, to tightly regulate endogenous gene transcription in the presence of blue light (126) (Figure 4A-I).

## REPRESSIBLE SYSTEMS FOR DYNAMIC CONTROL OF EPIGENETIC EDITING

Another strategy to chemically control gene expression for human cell reprogramming is based on SpdCas9 linked to VP192 activator and N-terminally fused to a dihydrofolate reductase (DHFR)-derived destabilization domain. The addition of the small molecule trimethoprim (TMP) temporally stabilizes the complex, thereby providing control over degradation of the DHFR fusion construct. However, this system has been shown to exhibit a degree of leakiness due to partial protein degradation (127) (Figure 4B-I).

Moreover, both the DHFR-TMP strategy and the MS2-VP64 system have been combined with the development of auxin-inducible degron (AID) technology. The AID system involves tagging Sp or SadCas9 with the auxin plant hormone-sensitive domain IAA17 and co-expression of the auxiliary protein TIR1. The administration of TMP allows for drug-tunable gene upregulation, which can be abrogated by the addition of auxin to the system as this results in rapid dCas9 protein proteasomal degradation (128) (Figure 4B-IV).

Lastly, the AID approach has been exploited to assess epigenetic memory and gene regulation in the chronic myelogenous leukemia cell line K562. Use of AID technology allowed temporal control of SpdCas9-p300 expression. The epigenetic editor was targeted to distal non-regulatory genomic regions, thereby reprogramming them into enhancer-like elements (i-Enhancer), by depositing H3K27ac marks, and induction of gene expression from the proximal promoter (129) (Figure 4B-II).

As an alternative to chemical approaches, interference and competition with CRISPR/dCas9 DNA recognition represents a valid tool to regulate its epigenetic editing activity. Since the CRISPR–Cas system is a natural bacterial defense against foreign invading elements, such as plasmids or bacteriophage infections (10), as a countermeasure, several phages have evolved to express proteins that block the CRISPR–Cas system. These proteins are called ‘anti-CRISPR’ or Acr proteins.

Alan Davidson's laboratory was the first to identify small inhibitory proteins specific to the type I CRISPR–Cas system, which does not include Cas9 proteins (130). The discovery of AcrII2 and AcrII4 proteins has resulted in successful inhibition of the widely used SpCas9 (131). The crystal structure revealed that AcrIIA4 inhibits SpCas9 activity by mimicking PAM, thereby blocking PAM recognition on the DNA by SpCas9 (132). For instance, demethylation of the *FMR1* gene promoter and its expression were maintained for at least 14 days after inhibition of SpdCas9-TET1 by the potent AcrIIA4 protein. However, the inhibition of DNA demethylation was irreversible (62).

To control AcrIIA4 expression, a miRNA-responsive AcrIIA4 switch has been designed to cell-specifically regulate dCas9-VPR-mediated gene activation (133). Similarly, miR-122-dependent knockdown of AcrIIA4 has been developed that resulted in SpdCas9-VP64 activity and thus luciferase expression in the hepatocellular carcinoma cell line HuH-7 (134) (Figure 4B-III).

Optogenetic control of AcrIIA4 is another option, based on fusion of the Acr protein with the photosensor LOV2 from *Avena sativa*, referred to as the CASANOVA system. In the presence of blue light, the AcrIIA4-mediated inhibition of SpdCas9-p300 is suppressed, thereby resulting in acetylation of the genomic loci of interest (135) (Figure 4B-V).

Finally, new anti-CRISPR proteins, such as AcrIIA2 and its more potent homolog AcrIIA2b, have been shown to be temperature-sensitive blockers of SpCas9 (136). These findings provided a basis for the design of synthetic small-molecule inhibitors, e.g., BRD0539 and BRD20322 (137), thereby further expanding the toolbox of CRISPR–dCas9 modulators.

## SPECIFICITY OF EPIGENOME EDITING TOOLS

The ultimate goal in molecular precision medicine is to target very specific disease-driving genes while minimizing the risk of off-target effects by not causing significant perturbations elsewhere in the genome. Compared to previous technologies, such as RNA interference (iRNA) methods (siRNA and shRNAs) (138), engineered proteins, particularly those based on TALE monomers and CRISPR/Cas platforms, have exhibited far superior genome-wide transcriptional specificity (30).

The binding specificity of genome editing tools depends primarily on the editing platforms employed and on the nature of the effector domains (EDs). Several studies, outlined in this section, have shown different or even partially contradictory results in regard to the exact mapping of DNA-binding events reported for each of these technologies. These discrepancies could, in part, be associated with the methods used to monitor off-target activities, which are determined by a number of ‘OMIC’ approaches, such as ChIP-sequencing (ChIP-seq), to detect the direct genome-wide binding events of the engineered proteins.

In the case of CRISPR/Cas nucleases (139–152) and base editors (153), several methods enable the mapping of indels and other genomic alterations in the genome. By contrast, for epigenome engineering tools, the genomic off-target DNA binding activities do not necessarily induce significant transcriptional changes and/or functional alterations in chromatin structure. As stated in some of the works below, ChIP-seq datasets, therefore, need to be integrated with RNA-sequencing (RNA-seq) and ideally with other techniques to assess DNA accessibility, such as DNase-sequencing (DNase-seq) or by the more recent ATAC-sequencing method (ATAC-seq) to fully assess the specificity of the epigenome engineering tools.

Early studies of ZF proteins fused with the KRAB domain suggested a high degree of transcriptional specificity genome-wide, as measured by DNA expression microarrays (154). More recently, Grimmer *et al.* conducted more comprehensive on- and off-target analyses on a 6ZF protein linked to KRAB, confirming the widespread binding of ZF platforms. Out of ∼6000 promoters bound, mapped by ChIP-seq, only ∼10% of these were differentially regulated (comprising 264 upregulated and 416 downregulated targets), as assessed by RNA-seq. Interestingly, the linkage of the KRAB repressor to the 6ZF arrays led to a ∼5-fold increase in binding sites, and the new sites were predominantly located outside of promoter regions (8), thus highlighting the influence of the effector domain in binding specificity, possibly via KAP1 recruitment. Moreover, the ZFs centrally located in the 6ZF array had strong but degenerated consensus-binding sites, as expected from the predicted specificities and the intrinsic degenerative binding of the ZF units.

In contrast to ZF proteins, Polstein *et al.* demonstrated that pools of four TALE-VP64 constructs targeting 17–18 bp in the *IL1RN*, *HBG1* and *HBG2* gene promoters were highly specific across the genome. ChIP-seq analyses unveiled between 4 and 31 off-target binding sites, although this did not result in significant transcriptional changes in off-target sites (37).

Another report suggested some non-specific DNA demethylation activity with TALE-TET1 fusions targeting 17–18 bp in the *KLF4* gene promoter, as observed by high-throughput bisulfite sequencing. The presence of off-target activities in the *HBB* locus was conceivably attributed to the pool of chimeric molecules residing in the nucleus without binding the cognate DNA sequence, although no significant changes in *HBB* gene expression were observed (58).

Similarly, TALEs comprising 17–20 repeats fused to LSD1 histone demethylase selectively targeted endogenous enhancers, as confirmed by ChIP-seq and RNA-seq. These constructs down-regulated gene expression in both the nearest upstream (*FAM18A*, *PLP2* and *ZFPM2*) and downstream (*ERMP1*) genes (113). Moreover, Amabile *et al.* achieved highly selective and durable epigenetic silencing by co-expressing TALEs individually fused to KRAB, DNMT3A and DNMT3L. In this case, the DNAme mapping and RNA abundance were determined by whole genome methylated DNA immunoprecipitation followed by deep-sequencing (MeDIP-seq) and RNA-seq, respectively. However, 10 off-target deregulated transcripts were reported experimentally, which were not computationally predicted (108). Furthermore, the single chimeric TALEs engineered by Mlambo *et al.* (N-KRAB-TALE-DNMT3A-Dnmt3l-C) have proven high specificity, with undetectable genome-wide perturbations, as confirmed by the superimposition of RNA-seq, ATAC-seq, and *in silico* prediction of off-target binding sites using PROGNOS software (109).

Inactive SpdCas9 protein in the absence of ED expression has been referred to as CRISPRi and exploited as gene repression tool, acting by interfering with the endogenous transcriptional activity. The DNA targeting specificity of dCas9 has been comprehensively assessed by ChIP-seq analyses, with off-target binding sites ranging from 10 to >1000 (155); from 26 to 6000 (156); or from 69 to 254 (157) across the genome, depending on the gRNA employed.

In contrast with CRISPRi, gene activation (CRISPRa) platforms have more consistently confirmed high precision in regulating target genes. For example, the SpdCas9-VP64 system demonstrated zero significantly upregulated off-target genes and one significantly down-regulated off-target gene, as monitored by RNA-seq (36). However, in another report, ChIP-seq identified 31 off-target binding sites for a pool of four SpdCas9-VP64 proteins, but again RNA-seq confirmed negligible changes in gene expression at off-target sites (37). Regarding the SpdCas9-MS2-SAM (46) and the SpdCas9-p300 (69) systems, only two off-target genes have been reported to date.

The specificity of CRISPR repressor systems (CRISPRr) revealed similar results to that of activation platforms. The SpdCas9-KRAB repressor has been particularly studied, and demonstrated high on-target specificity, as evaluated by RNA-seq, for the targeting of a reporter gene (the *GFP* gene driven by the SV40 promoter) (30) or the endogenous HS2 distal enhancer regulating the *HBE1*, *HBG1*, *HBG2*, and *HBB* downstream globin genes. In the latter study, non-significant off-target perturbations were detected by RNA-seq, ChIP-seq for the detection of H3K9me3, and DNase-seq for mapping of accessible chromatin regions (85). Interestingly, and in contrast to ZF proteins, the linkage of KRAB to SpdCas9 did not alter the predicted specificity of DNA binding by dCas9–gRNA complexes (85,157).

Small-scale analysis based on ChIP-qPCR for histone modifications such as H3K4me2, H3K9me3, H3K27me3 and H3K27Ac has confirmed the high specificity of NmedCas9 from *Neisseria meningitidis* engineered with LSD1 targeting the *Tbx3* distal enhancer. However, gene expression microarray data revealed 174 differentially expressed genes, albeit not exceeding a two-fold change, thus suggesting that limited off-target effects occurred (114). Moreover, and similarly to TALEs, even pools of SpdCas9 proteins separately fused to KRAB, DNMT3A and DNMT3L demonstrated high specificity, with 14 off-target transcripts deregulated and one DMR detected across the genome. These off-target activities were not computationally predicted by the sequences of gRNAs used (108).

It is worthy to note that, for synthetic induction of DNAme by dCas9 systems, Lin *et al.* conducted whole-genome bisulfite sequencing (WGBS) to investigate the specificity of SpdCas9 proteins fused to either DNMT3A or DNMT3B, identifying >1000 off-target DMRs, with hypermethylated regions mapping in promoter regions, 5′ untranslated regions, CpG islands, and in DNase I hypersensitivity sites. Interestingly, the hypomethylated CpG sites mapped in repetitive sequences, such as Alu and LINE1 interspersed elements (105).

Conversely, the SpdCas9-SunTag lentiviral system, which recruits multiple DNMT3As, identified only 35 hyper- and 30 hypomethylated off-target CpG sites in addition to the expected on-target hypermethylation in the *HOXA5* promoter by reduced representation bisulfite sequencing (106). These off-target activities may be associated with the constitutive expression of the long isoform of DNMT3A, which potentially could bind to and/or be recruited into the DNA in a gRNA-independent manner. Pflueger *et al.* more recently confirmed the specificity of the SpdCas9-SunTag-DNMT3A-CD system in transient transfections by ChIP-seq, evidencing 13 off-target peaks, three of which exhibited an increase in DNAme mapped by targeted bisulfite PCR sequencing (bsPCR-seq) (107).

In summary, both Cas9 and TALE systems demonstrate a degree of off-target activities, which can be exacerbated by certain EDs, in particular DNMTs. Researchers should carefully map and determine potential non-cognate target sites, ideally by integrating several ‘OMIC’ and computational platforms. However, binding to off-target sites does not necessarily produce significant transcriptional perturbations in associated genes since the probability of binding to sequences mapping in proximity to regulatory regions, such as TSSs and enhancers, is relatively low. Validation methods, such as the engineering of multiple DBDs targeting the same regulatory region, as well as functional rescue experiments, are, therefore, recommended to fully validate the application of these tools in cells, tissues, and organisms.

### The origins of off-target activities

Potential off-target activities of genome engineering tools have been attributed primarily to the DNA base-pairing specificity of the DBD, for example the ZF and TALE domains, or the gRNAs interacting with genomic sequences for Cas9 and Cas12a proteins.

For protein-based modules, early studies demonstrated that the number of domains or repeats assembled in ZFPs or TALEs increased the specificity by expanding the number of DNA contacts and, therefore, reducing the frequency of DNA binding events genome-wide. For example, 3ZF proteins, which only recognize 9 bp, are expected to recognize thousands of sites in a complex genome, such as the human genome (158). By contrast, multimodular 6ZF proteins, which recognize 18 bps, were anticipated to potentially regulate single genes (159). Similarly, TALEs comprising >18 repeats have the potential to regulate single genes (160). Moreover, the structural characteristics of the binding of individual domains as well as the specific arrays assembled play a fundamental role in DNA recognition (7). For instance, the N- and C-terminally located ZFs have shown decreased selectivity, with some ZFs not contributing to consensus sequence binding or not binding to the DNA at all (161), while the extension of the linkers between ZFs provided an enhanced DNA-binding affinity (162).

On the other hand, some residues mapping in key alpha-helical positions of the ZF, such as –1, +3 and +6, can tolerate different amino acids side chains and still bind DNA (163,164). This redundancy is at the basis of non-cognate off-target effects observed with ZFPs, which can be predicted computationally (165–169). In contrast to ZFs, the recognition of DNA by TALE repeats is more selective, with each repeat specifying a single base pair in a one-to-one recognition fashion, thereby making TALEs improved tools for recognizing unique sites in complex genomes (9).

In the case of RNA-guided platforms, such as Cas9 systems, the recognition of the PAM sequence dictates the initial Cas9 binding to the DNA, while the 20 nt target site complementary to the DNA is essential for binding specificity. Within this complementary sequence, the specificity is mainly conferred by the 3′ end of the gRNA, referred to as ‘seed region’, which comprises 10–12 nt upstream of the NGG PAM sequence (170). Therefore, mismatches in the core of the gRNA seed region (specifically the first 5 nt upstream of the PAM sequence) severely compromise the binding to the target DNA and consequent cleavage of SpCas9 proteins. In contrast, more distal mismatches can potentially be tolerated, thereby giving rise to potential off-target effects (156,157).

Aside from genomic sequences that contain mismatches with the gRNA, off-target binding loci can also originate from targeted DNA sequences that either contain insertions (a DNA bulge) or that have nucleotide deletions (generating a gRNA bulge) relative to the designed gRNA sequence (171). Importantly, ‘hot-spots’ that are rich in PAM motifs, such as CpG islands, may act as decoys for SpdCas9, thereby decreasing on-target specificity (157).

Regarding Cas12a systems, recent studies have characterized the thermodynamic determinants that influence the DNA binding of FndCas12a from *Francisella novicida*, including an extended PAM sequence that increases the surface of DNA binding. Moreover, the platform is more permissive therefore more biased towards dT:rU and dG:rG than to dA:rA and dC:rC mismatches (d = DNA, r = crRNA guide). Lastly, the system tolerated mismatches occurring after the 17^th^ nt on the crRNA distal region, and up to three mismatches within the 6 nt of the seed region (172).

In addition to the nature of the DBDs, other factors can influence off-target activities. In contrast to catalytically active Cas9, dCas9 proteins are artificially linked to one or more EDs, potentially influencing the recruitment of the resulting fusions in the genome. Notably, the catalytic domain of DNMTs fused to the dCas9 protein induced *de novo* DNAme activities in both a gRNA-dependent and a gRNA-independent manner (105). The latter generated unspecific DNAme effects, aggravated by the absence of a linker between SpdCas9 and the catalytic domain of DNMTs as well as by an elevated presence of the chimeric fusion proteins in the nucleus when overexpressed. SpdCas9-SunTag systems, which recruit various DNMT3A domains in the same genomic site, could minimize off-target activities as compared to direct SpdCas9-DNMT3A fusions (107).

The ChIP-seq analyses conducted by Wu *et al.* and similarly by Kuscu *et al.* have shown conspicuous non-specific DNA binding events by inactive SpdCas9, particularly in open chromatin regions, thus highlighting that the local chromatin structure and the chromatin context can strongly influence the binding of SpdCas9 to its nucleic acid target site (155,156). Similarly, several works reported the co-delivery of multiple activators targeting the same gene promoter, such as TALEs-VP64 for the regulation of *VEGF-A*, *NTF3* promoters (29) and *CEACAM5*, *KLK3*, *IL1RN* and *ERBB2* (33); TALEs-VP64 in combination with ZFs-VP64 for targeting the tumor suppressor genes *MASPIN* and *REPRIMO* (54); and TALEs-p65 for the upregulation of the *miR-302/367* cluster's gene promoter (29). These studies concluded that combinations of activators resulted in a higher on-target transcriptional specificity relative to that of single agents, resulting in synergistic gene regulation. The basis of this increased transcriptional specificity stems from the synergistic interactions between the platforms, requiring the expression of very small amounts of each regulator. Lastly, artificial proteins also exhibited pharmacological synergisms with combinations of chromatin remodeling drugs, such as 5-aza-2′-deoxycytidine (Decitabine) and suberoylanilide hydroxamic acid, i.e., SAHA (Vorinostat) for the regulation of targeted genes in heterochromatic contexts (173). Of note in this regard, off-target binding sites that are not in proximity to functional regulatory regions, such as TSSs and enhancers, are highly unlikely to result in off-target activities (45,52,174). Interestingly, in another work, engineered ZFs linked to the KRAB domain were able to activate, and not repress, the targeted *OCT4* promoter, marked by DNAme in cancer cells. Again, this outlines the influence of the chromatin microenvironment on the outcome of gene regulation (175).

In summary, these works suggest that the origins of off-target specificity are not only intrinsically dependent on the platforms used for epigenetic engineering, but also driven by ‘extrinsic’ factors. These include genomic sequence variations, the genomic context, the chromatin structure and chromatin microenvironment (e.g., the presence of cell-type-specific co-factors and epigenetic regulators) at targeted loci, which can highly influence both the binding and the efficiency of the regulation of different epigenetic editing platforms.

### Strategies to improve specificity

To improve specificity, efforts have been directed at re-engineering the DBD or DNA-binding activity of artificial transcription factors. Several strategies, outlined below, focus on: 1) re-engineering and molecular evolution of DBDs/gRNAs; 2) variations on the gRNA design; 3) synthetic Cas9 variants; 4) Cas9 and Cas12a orthologues; 5) cascade systems (CRISPR Class 1 systems); 6) ED recruiting technologies for CRISPR systems; 7) spatiotemporal control of DBDs and EDs.

1. Re-engineering and molecular evolution of DBDs and gRNAs
  In the case of ZFs (159) and TALEs (160), increasing the number of domains or repeats to recognize 18 or more bps has been shown to enhance the DNA binding specificity by potentially recognizing single sites in the genome. Several online tools are available to the scientific community for the assembly of both ZFs (165–168,176,177) and TALE repeats (178–185). As regards ZF arrays, re-engineering of ZF domains using molecular modelling and by selection of ZF variants by phage display methods has been shown to enhance the DNA-binding selectivity (186–189), with some of these ZF arrays entering clinical trials (190–192).
  The binding specificity of TALEs has also been improved by several methods, including: (i) the identification of the diresidue asparagine-histidine (NH), as a more stringent guanine-specific repeat-variable diresidue (RVD) (193); (ii) the discovery of new repertoires of RVDs (194,195); (iii) the incorporation of bivalent cations, i.e., Mg^2+^ or Ca^2+^ in the binding reaction *in vitro* (196). (iv) Lastly, the histidine-aspartic acid (HD) repeat unit is potentially allele-specific, discriminating single-nucleotide polymorphisms (SNPs), e.g., the C/G SNP (185).
  With respect to CRISPR/Cas systems, bioinformatic pipelines and web-based algorithms are available to provide guidance for optimal gRNA design, thereby minimizing predictable off-target activities (170,197–209). The designed gRNAs are highly unlikely to generate off-target effects, as experimentally validated by the verification of *in vivo*off-targets (VIVO) method. In this strategy, the potential off-target cleavage sites from the gRNA/SpCas9 endonuclease ribonucleoprotein (RNP) system are first identified *in vitro* on the genomic DNA extracted from an animal model, employing the highly sensitive circularization for *in vitro*reporting of cleavage effects sequencing method (CIRCLE-seq) (148). These identified off-target loci are subsequently validated for indel mutations by targeted amplicon sequencing performed on the same genomic DNA isolated from the animal tissue experimentally treated with the same gRNA/SpCas9 endonuclease system delivered by adenoviral vectors (150).
  Importantly, the cell-type-specific and personalized SNPs need to be taken into account when assessing off-target specificities as outlined by the highly sensitive CIRCLE-seq method. In this study, Tsai *et al.* overlapped the detected off-target sites for six gRNAs targeting cell lines with the genotypes of individuals from the 1000 Genomes Project Consortium (210). The authors found that indeed genetic variations enhanced the number of off-targets by increasing the range of mismatches. Importantly, a single SNP is expected in ∼69% of SpCas9 off-target loci in the human genome (148). This method can be particularly important to generate personalized specificity profiles in order for gene therapy to be translationally applied to patients. In addition to SNPs located in DNA regions complementary to the gRNA as well as the PAM, for epigenome editing it is crucial to map the location of enhancers, promoters, and other regulatory elements. Several comprehensive public resources provide information on chromatin accessibility, nucleosome positioning, DNAme (presence of CpG islands), enhancers and super-enhancers (NIH Roadmap Epigenomics (211), ENCODE (212,213) and FANTOM5 (214,215) projects).
2. Variations on the gRNA design
  Multiple strategies have been developed to improve the DNA binding specificity of the gRNAs. First, regarding the seeding region, Fu *et al.* have shown that truncated gRNAs <20 nt (17 or 18 nt) can still bind efficiently to the cognate DNA target site while reducing off-target activities by 5000-fold for particular target sites (216). Further shortening of gRNAs down to 14 nt (referred to as ‘dead’ gRNAs, as they abolish the Cas9-mediated DNA cleavage while maintaining the DNA binding) are less tolerant to mismatches when guiding SpCas9-VPR to reporter genes. These shorter gRNAs did not increase unappreciated off-target activities, as assessed by RNA-seq (217).
  Lastly, in addition to the length of the gRNAs, hairpin secondary structures engineered on the spacer region of either gRNAs (Cas9) or crRNAs (Cas12a) can increase the specificity of the gRNAs by thermodynamically increasing the instability with their off-target sites (218).
3. Synthetic Cas9 variants
  Repertoires of SpCas9 variants have been rationally engineered to improve on-target genome editing specificity, by designed point mutations (219–222) or by directed evolution screenings performed in yeast (223) and bacteria (224). Examples of both are the enhanced eSpCas9 (K810A, K1003A, R1060A) (219), the high-fidelity SpCas9-HF1 (N497A, R661A, Q695A, Q926A) (220), followed by the hyper-accurate variant Hypa-SpCas9 (N692A, M694A, Q695A, H698A) (221), and the evolved evoSpCas9 (M495V, Y515N, K526E, R661Q) (223), all delivered in cells as plasmid DNA. The Sniper-SpCas9 (F539S, M763I, K890N) (224) and the high fidelity HiFi-SpCas9 (R691A) (222) variants have been delivered in a preassembled RNP format, the latter for the treatment of sickle cell disease.
  Additional SpCas9 variants have been generated to increase DNA targeting specificity, as is the case of dCas9-VQR (D1135V, R1335Q, T1337R), -EQR (D1135E, R1335Q, T1337R) and -VRER (D1135V, G1218R, R1335E, T1337R). These are SpCas9 derivatives that harbor the PAM-interacting (PI) domain mutagenized via bacterial-selection based screening (225) or through phage-assisted continuous evolution (226). All these variants possess altered and novel PAM recognition specificities.
4. Cas9 and Cas12a orthologues
  The specificity of epigenetic editing by Cas9 and Cas12a systems can be maximized by taking advantage of natural variants. Specifically, the two type II-C Cas9 compact orthologues, NmeCas9 from *Neisseria meningitidis* (1082 aa) (227,228) and CjeCas9 from *Campylobacter jejuni* (984 aa) (229) are naturally hyper-accurate. This enhanced specificity is attributed to the presence of either longer gRNAs (24 nt for Nme and 22 nt for Cje, compared to the 20 nt gRNAs for SpCas9 (1368 aa)) or to more stringent and larger PAM sequences. Moreover, these constricting PAM requirements highly reduce the frequency of on-target sites in the genome. Furthermore, among the available arsenal of small Cas9 orthologues, SaCas9 (1053 aa) represents a multi-turnover enzyme, a fact attributed to its weaker affinity for gRNAs. This is in contrast to the low-rate kinetics observed for SpCas9. Such high turnover could be potentially advantageous for enhancing the efficiency and specificity of CRISPR systems when employing SadCas9 orthologues for epigenetic editing (230).
  It is worth noting that Cas12a orthologues (AsCas12a more so than LbCas12a) exhibit higher DNA binding specificity than SpCas9, evaluated by the Breaks Labeling In Situ and Sequencing (BLISS-seq), which is a sensitive method for detecting genome-wide off-target activity (149). Additionally, the novel CeCas12a orthologue from *Coprococcus eutactus*, with its more stringent non-canonical PAM recognition, provides another potentially more specific toolkit for epigenome engineering (231). Lastly, another high-fidelity version of Cas12a is the enhanced enAsdCas12a-HF1 variant from *Acidaminococcus sp*. orthologue, while enAsdCas12a has already been fused for gene activation by a fusion with the VPR activation domain (232).
5. Cascade systems (CRISPR Class 1)
  Beyond the canonical Class 2 CRISPR–Cas systems (Cas9 and Cas12a), Pickar-Oliver *et al.* have recently engineered the multi-component Class 1 CRISPR-associated complex for antiviral defense ‘Cascade’ systems from *Escherichia coli* (EcoCascade) and from *Listeria monocytogenes* (LmoCascade). Similarly to Cas systems, Cascade proteins can be fused with EDs such as p300 and the KRAB for specific gene activation and repression, respectively.
  These novel parallel approaches, employing longer crRNAs of 32 nt (EcoCascade-p300) and 36 nt (LmoCascade-KRAB) in length, respectively, demonstrated high genomic selectivity (233). For instance, VPR fusions to Cascade systems from *Pseudomonas aeruginosa* (PaeCascade-VPR) guided by 32 nt crRNAs are capable of activating gene transcription of therapeutic genes such as *HBB* and *HBG* to treat β-thalassemia, without predicted off-target activities. This recent technology is very sensitive to crRNA-DNA mismatches, including those very distal to the PAM sequence (234). Despite this high specificity, the delivery *in vivo* of such bulky multi-modular complexes represent a potential obstacle for subsequent translational applications. Furthermore, the cytotoxicity and immunogenicity of these emergent tools also need further investigation for entering clinical pipelines.
6. ED recruiting technologies for CRISPR systems
  In addition to the nature of the gRNAs, the specific strategy employed to recruit the EDs to dCas9 can highly influence the binding selectivity. For example, direct fusions of SpdCas9-DNMT3A may be more prone to generate off-target activities (105) compared to recruiting-based platforms, such as SpdCas9-SunTag-DNMT3A, where multiple enzymes are simultaneously engaged in the same region of the genome (107). In this system, even the delivery of a single gRNA elicits very effective epigenetic editing (106).
  Similarly, several recruiting platforms have been developed for the delivery of DNMTs or other enzymes by the aptameric, Casilio, and SunTag technologies, in order to boost on-target versus off-target DNAme activities. Moreover, recruitment systems can also bypass the tiling of promoter regions with many gRNAs, thus potentially lowering unintended off-target effects.
7. Spatiotemporal control of DBDs and EDs
  Another strategy to improve the on-target activity of genome engineering tools is to tightly and dynamically control the expression of the platforms. This is particularly important for epigenetic domains that have intrinsic enzymatic activity, such as the DNMTs. For example, McDonald *et al.* observed significantly reduced off-target effects when SpdCas9-DNMT3A-CD direct fusions were induced by doxycycline (Dox) relative to that of the same fusions expressed in a constitutive system (97). Given that Dox-inducible systems rely on promoter-driven transcriptional regulation, the remaining dCas9 gene expression upon Dox removal can potentially be a concern for particular biomedical applications involving dCas9 systems. Anti-CRISPR peptides, e.g., AcrIIA4 can strongly inhibit Cas activities ‘at command’ for more rapid and tunable control of epigenome editing in mammalian cells (235).
  In general, methods that ensure both spatial (cell-type-specific) and temporal control of epigenetic editing will be crucial for the development of more selective epigenome engineering tools. While Cas and TALE backbone systems are present naturally in bacteria and plant pathogens, respectively, mammalian cells already utilize several endogenous DBDs/TFs to precisely control cell-type-specific cell fates. Endogenous DBDs/TFs are often tightly spatiotemporally controlled by networks of TFs, which ensure that the right regulator is expressed at the proper threshold (expression levels) and within a given tissue, and in specific cell types. Similarly, future engineered epigenome editing technologies may not only consider on-target ‘genomic’ selectivity, but also ‘cell type’ selectivity. This could in the future be achieved by engineering artificial/synthetic networks of TFs (236–238), by using tissue-specific regulatory promoters, and by targeting delivery systems with cell-type-specific ligands/receptors.
  In summary, both TALE and CRISPR platforms have been developed to modify single genes with minimal, in some cases negligible, off-target activities. This can be achieved by modification or direct evolution of the proteins, by using different variants of Cas proteins (orthologues, high-fidelity variants), by modifications of gRNAs (shorter spacer regions, incorporation of secondary structures), and by the recognition of more specific and stringent PAM sequences. Moreover, the choice of the method/s to assemble different effectors as well as the spatiotemporal control of the epigenetic editing tools are important, particularly when manipulating DNAme. Lastly, ‘cell type’ in addition to the canonical ‘genomic’ specificity should also be taken into account when designing an effective expression strategy for epigenomic editing *in vivo*.

## STABILITY OF THE EDITED EPIGENETIC STATE

An ultimate goal of epigenetic editing is the maintenance of gene expression patterns over cell divisions, which can be achieved by engineering of several epigenetic domains. Some epi-marks, such as DNAme (239) and H3K9me3 (240), are faithfully transmitted during DNA replication (241,242); thus *de novo* induction of these marks by engineered domains can be expected to be ‘read’ and ‘maintained’ by the endogenous epigenetic regulators during DNA replication.

In regard to gene repression, several works support the notion that DBDs linked to the CD of DNMT3A, such as ZF-DNMT3A-CD (80,81,92) and dCas9-DNMT3A-CD (88,96) or even DBDs-KRAB, could lead to a window of maintenance of gene repression, such as ZFs targeting the *MASPIN* (80) or the *SOX2* (79,81) gene promoter or SpdCas9 targeting enhancers (85).

An exciting prospect of epigenetic editing is the capacity of these toolkits to potentially modify chromatin with very long lasting effects, ideally causing a permanent reprogramming. However, the temporal window of ‘epigenetic memory’ reported in native chromatin contexts varies between different studies, from 5 to 10–15 days for both DNMT3A-CD (88,92,96,243) and KRAB (30,45,85,88,244) to up to 100 days in an inducible ZF-DNMT3A-CD system in breast cancer (81); at least 31 days employing 6ZFs-DNMT3A-CD targeting the *MASPIN* TSG promoter in breast cancer (80), or ∼168 days employing SadCas9-KRAB in the liver of adult mice (245). In contrast, another study deploying ZFs linked to DNMT3A-CD targeting the *VEGF-A* gene promoter suggest that the poor maintenance of gene silencing (10–15 days) may be due to the transient duration of the epigenetic editing stimulus (243).

These aforementioned studies point to the notion that, while achievable, durable epigenetic editing relies on the chromatin context and the nature of the targeted gene. For example, targeting TFs, such as *SOX2*, which are often regulating epigenetic modifiers themselves, could be a good strategy for reinforcing the initial effect of the synthetic regulators (79–81). This, in turn, could be related to several factors such as the specific combinations of epigenetic effectors used and to the epi-marks and the abundance of specific co-repressors, and/or other epigenetic modifiers at particular regulatory regions.

Lastly, the outcomes of gene silencing may also be highly dependent on the technology used for delivery. More persistent entities, such as lentiviral vectors might be more efficient at imprinting and maintaining the desired epigenetic marks compared to ‘hit-and-run’ transient delivery vehicles, including adenoviruses, adeno-associated viruses (AAVs), liposomes, or other nanotechnology vehicles. However, the manifestation of potential higher off-target activities, when a genomic integration choice is pursued, has to be considered carefully and addressed by using newer and more reliable and precise inducible systems.

Sustained gene repression of highly overexpressed targets, such as oncogenic drivers, represents a great challenge for epigenetic editing technologies, particularly when using transient vectors. Several works have demonstrated that the assembly of multiple epigenetic domains could be highly effective for increasing the longevity of the repression state, even when using ‘hit-and-run’ transient delivery methods. For instance, the DNMT3A-CD (DA) and DNMT3L (DL) have been combined with either KRAB (K) (108,109) or Ezh2 (E) (112) in the context of TALEs and SpdCas9 platforms, and these entities exhibited long-lasting gene silencing. This enhanced epigenetic longevity ranged from ∼30 days (for the combination of SpdCas9-DA, SpdCas9-DL and SpdCas9-K fusions) to ∼50 days (for the combination of TALE-DA, TALE-DL, and TALE-K fusions) (108), ∼21 days (for the K-TALE-DA-DL single fusion) (109), and ∼50 days (for the combination of DA-SpdCas9 and E-SpdCas9 fusions along with overexpressed DL) (112). Overall, these studies suggest that strategies combining several distinct effectors might be more effective at maximizing the epigenetic and transcriptional ‘memory’ of the repressive state.

It is important to note that the majority of studies so far had mostly focused on the synergisms between repressive machineries, although ‘on’ epi-marks (associated with gene activation) may still be present in the targeted genes. In fact, a genome-wide study with ZF-DNMT3A-CD found that genes that quickly ‘relapsed’ from *de novo* DNAme induced by the ZFs were marked by H3K4me3 (246). Thus, future works should evaluate a concomitant targeting of ‘on’ and ‘off’ epi-marks for effective and more persistent epigenetic editing.

A few works have explored combinatorial ED strategies to maximize the durability of the activation state, although much more research in the arena of gene activation needs to be conducted to identify long-lasting and durable gene activators. Cano-Rodriguez *et al.* have found that the combination of two KMTs targeting histone H3, PRDM9 (inducing H3K4me3) and DOT1L (inducing H3K79me), fused to Dox-inducible SpdCas9 proteins, resulted in sustained (20+ days) gene re-expression of hyper-methylated gene promoters (76). Another example is the co-targeting of synergistic EDs by different orthologues, such as TET1-CD-SadCas9 and VPR-SpdCas9, which resulted in durable reactivation of gene expression (∼30 days) (19) compared to the ∼14-day gene re-expression with SpdCas9-TET1-CD (62).

Furthermore, the combination of TET1-CD (catalyzing DNA demethylation) with Base excision repair (BER) enzymes (e.g., GADD45A or NEIL2) using the SpdCas9 Casilio recruiting system (67), or TET1-CD and VP64, simultaneously recruited by an optimized single gRNA/SpdCas9-SunTag platform, also enhanced the upregulation of several hypermethylated genes in a lung adenocarcinoma cell line (247). It might be of interest to determine whether these combinations of enzymatic activities or synergistic epigenetic effects could also enhance the longevity of the activation state in promoters marked by DNAme.

In general, the simultaneous writing and erasing of mutually exclusive epi-marks that co-exist at particular promoters (e.g., H3K4me3 and 5mC, or H3K9ac and H3K9me3) may be a key requirement for more sustained synthetic epigenetic manipulation. As more becomes known regarding the molecular epigenetics of most cell types and tissues, tailored therapeutic strategies may be devised whereby epigenetic regulators are combined to achieve more effective and sustained ‘reprogramming’ of targeted cells.

## TARGETED DELIVERY SYSTEMS FOR dCas9-MEDIATED EPIGENOME EDITING *in vivo*; PRE-CLINICAL, AND CLINICAL STUDIES/TRIALS

Personalized medicine by epigenome editing requires precise *in vivo* delivery of the molecular tools to the proper cell types and tissues to restore the altered epigenome in these cells. Several strategies have been pursued for gene therapy. These comprise viral-based approaches, mainly adeno-associated viruses (AAVs), and non-viral-based approaches, which predominantly involve targeted cationic lipid nanoparticles (NPs) and polymers.

For therapeutic epigenome editing, AAVs pose safety concerns due to pre-existing immunity towards them in the majority of the human population (248). Moreover, the eleven well-characterized AAV serotypes that dictate the diverse and selective tissue tropism may not be suitable for cancer treatment as neoplastic cells tend to exhibit abnormal cell surface features. Therefore, the generation of chimeric vectors with low immunogenicity and tailored for cancer cell specificity might prove to be more useful tools for gene delivery. Viral integration into the host genome will nonetheless still result in sustained expression of dCas9/12a protein fused to EDs, which could potentially lead to unintended off-target effects. Additionally, the viral titers required to achieve therapeutic levels are exponentially higher than the clinically approved levels. Lastly, the small packaging capacity of AAVs (∼5 kb) constitutes another limitation, particularly for the delivery of bulky EDs or multiple EDs linked to the already quite sizeable dCas9 protein.

To circumvent the AAV payload limit, SpdCas9 (4.2 kb) with an intein-mediated split at its disordered linker (V713–D718) can be used to facilitate incorporation of the VPR domain (1.6 kb) into various AAV9 vectors. No widespread cellular damage has been noted *in vivo*, although it did elicit immunological reactions. Additionally, since this system relies on the trans-splicing machinery, it might not be the ideal approach to attain sufficiently high levels of gene activation *in vivo* to induce physiologically relevant phenotypic changes (249). To overcome this limitation, a dual-AAV9 vector system has been devised to epigenetically remodel and thus transcriptionally activate genes in mouse models of human diseases, including regenerative medicine applications for muscular dystrophy, diabetes, and acute kidney disease. The system comprises SpCas9 and a truncated 14-bp dead MS2 dgRNA that prevents the active Cas9 nuclease from creating double-stranded breaks, yet that is capable of binding to the DNA and recruiting MS2 coat protein-p65-HSF1 for robust target gene activation *in vivo* (250). A therapeutically safer AAV9 has also been employed to separately deliver the considerably smaller SadCas9 fused to 2xVP64 and gRNAs to upregulate the compensatory modifier gene *Lama1* in a mutation-independent fashion in a mouse model of congenital muscular dystrophy type 1A (251).

Moreover, a dual-vector approach has been devised for programmable long-term gene silencing. AAV8 vectors expressing SadCas9 fused to KRAB and a gRNA targeting the *Pcsk9* gene, respectively, were systemically administered in a wild-type adult mouse model. Transcriptional repression of *Pcsk9*, a gene responsible for cholesterol homeostasis, was maintained for 24 weeks after a single treatment in post-mitotic tissue, with a moderate host response and coincidental liver toxicity. This was primarily due to SadCas9-KRAB expression rather than the AAV8 used as gene vector (245), given the high presence of antibodies against Sa and SpCas9 proteins in the adult human population (252).

As an alternative to the dual vector system, researchers have engineered the all-in-one mini-dCas9 system, which can be incorporated into a single AAV vector. It consists of a compact version of transactivators based on VPR domain C-terminally fused to a downsized variant of SadCas9 along with an optimized gRNA expression cassette to maximize gene activation and for potential delivery *in vivo* (253).

In cancer therapy, the novel telomerase-activating gene expression (TAGE) system exploits the synergy of dCas9-VP64-mediated gene activation and Cas9 endonuclease activity to specifically target cancerous cells *in vivo* (254). As non-cancerous cells do not exhibit telomerase activity, the telomere injury and consequent cell apoptosis caused by the TAGE approach has been found to only occur in oncological conditions. However, multiple recombinant AAVs have to be used, which may limit virus dosage control and thus its applicability in clinical studies. Nanoparticle carriers can be harnessed to facilitate the translation of these gene therapy techniques to the clinic.

Nanotechnology represents a valid alternative to AAV-based delivery systems. This is due to the flexibility of the scaffolds, which allows large cargoes to be accommodated. Furthermore, the use of specific ligands attached to NPs makes this non-viral delivery approach more targetable for cancer treatment applications. Finally, PEGylation and other biodegradation-related strategies may render the NPs immunologically inert or reduce their immune clearance, stimulate cell internalization or endocytosis, and promote endosomal escape.

The use of cyclic RGD-functionalized dendritic polymers has enabled targeted activation of TSGs via SpdCas9-VPR in a mouse model of breast cancer. A long-lasting therapeutic effect with negligible tissue toxicity was achieved after intravenous injection of the plasmid DNA polyplex (255). Furthermore, sufficient levels of endogenous tumor suppressor *miR-524* upregulation have been achieved in a mouse model of triple-negative breast cancer to result in a therapeutic effect. Multistage delivery nanoparticles (MDNP) were developed to efficiently tumor-target SpdCas9-VP64 and gRNAs as plasmid DNA polyplex via systemic administration. MDNPs have been intentionally designed to bypass physiological barriers, such as instability in the circulatory system and dissociation in the tumor microenvironment, followed by internalization into tumor cells (256).

In contrast to plasmid DNA, neither transcription nor translation are required for RNP delivery. Moreover, cytosolic DNA has been shown to induce host immune responses (257). Therefore, the delivery of RNPs may lead to a cell-cycle independent, faster, more effective and safer therapeutic effect.

*In vivo* delivery of the SpdCas9-VPR protein complexed with gRNAs that targets and upregulates the *HGF* gene (a hepatocyte growth factor essential for liver regeneration) led to a therapeutic effect in a mouse model of liver damage. The authors generated this novel genome editing with designed extracellular vesicles (GEDEX) system to mediate targeted transfer of RNPs based on extracellular vesicles produced and released by HEK293 cells (258).

## CONCLUSIONS AND PERSPECTIVES

Epigenome engineering has enormous potential as an innovative treatment for many human diseases, including cancer and neurodegenerative diseases. By strategically combining multiple different epigenetic effector domains and enzymes to rewrite the epigenome in a sustained manner, epigenetic therapy has a promising niche in a wide variety of human diseases. With the help of state-of-the-art genomic technologies, personalized medicine will also be a reality for many patients, minimizing side effects and thus improving overall survival. Nevertheless, many aspects, including off-target effects and ethical concerns, need to be addressed to extensively employ epigenome editing clinically. In light of the greater long-term safety of epigenome engineering *versus* genome editing, the former being potentially reversible and the latter potentially prone to unattended and more irreversible changes in the DNA sequence, epigenetic editing approaches hold considerable promise for applications in a clinical setting. To date, there have only been clinical trials based on the use of the endonuclease Cas9. The ‘promise’ of epigenetic memory based on the faithful transmission of some epigenetic marks also provides avenues for long-lasting and durable therapeutic effects. Moreover, the success of epigenetic engineering strategies will be highly dependent on the collateral development of safe, immune-inert, and targeted delivery systems to enable dCas9/gRNA expression in specific cell types, such as cancer cells and other diseased tissues.

## Supplementary Material

gkaa1000_Supplemental_Files
