# Supplementary material for: Epigenome engineering: new technologies for precision medicine
Source: Nucleic Acids Res. 2020 Nov 16;48(22):12453–82. doi: 10.1093/nar/gkaa1000 (PMC7736826; doi:10.1093/nar/gkaa1000)
Supplement: gkaa1000_Supplemental_Files [file gkaa1000_supplemental_files.zip › Supplementary Table S1.pdf]

Supplementary Table S1. Chromatin modifications, writers and erasers of epigenetic marks for gene transcriptional activation

Chromatin	Modifications for gene transcriptional ACTIVATION	Genomic Location	Epigenetic Enzymes (Writers)	Epigenetic Enzymes (Erasers)
**DNA**	Low or no CpG methylation	Active promoters and active or bivalent enhancers (1)	**DNMTs**<br>1 (maintenance) (2)<br>3A and B (*de novo*) (2,3)<br>3L (cofactor) (4)	**DNA demethylases**<br>TET1, TET2, TET3 (5,6)<br>TDG (7)
**Histone H2B (tail)**	H2BK5me1	Active promoters, downstream of the TSSs (8)	**KMTs**<br>Unknown	**KDMs**<br>Unknown
	H2BK5ac	Active promoters (9)	**KATs**<br>3A (CBP) (9)	**HDACs**<br>6 (10)
	H2BK20ac	Active promoters and enhancers (11)	**KATs**<br>Unknown	**HDACs**<br>Unknown
	H2BK120ub1	Promoter regions (12)	**Ubiquitin E3 ligases**<br>RNF20 and RNF40 (BRE1-A and BRE1-B) (12)	**Deubiquitinating hydrolases (DUBS)**<br>USP44 (13)
**Histone H3 (core and tail)**	H3K4me1 (tail)	Active enhancers and gene body (14)	**KMTs**<br>2A, B, C, D (MLL1, 2, 3, 4) (15,16)<br>2F and 2G (SETD1A and B) (17,18)<br>3C (SMYD2) (19)<br>7 (SETD7) (20)<br>8B (PRDM9) (21)	**KDMs**<br>1A and 1B (LSD1 and 2) (22,23)<br>5B (JARID1B) (24)<br>NO66 (JMJD9) (25)
	H3K4me2 (tail)	Downstream active TSSs (1,14)	**KMTs**<br>2A and 2B (MLL1 and 2) (15)<br>2F and 2G (SETD1A and B) (17,18)<br>3E (SMYD3) (26)<br>8B (PRDM9) (21)	**KDMs**<br>1A and 1B (LSD1 and 2) (22,23)<br>5A-D (JARID1A-D) (27-30)
	H3K4me3 (tail)	Active or bivalent TSSs (poised genes) (14)	**KMTs**<br>2A and 2B (MLL1 and 2) (15)<br>2F and 2G (SETD1A and B) (17,18)<br>3E (SMYD3) (26)<br>8B (PRDM9) (21)	**KDMs**<br>2B (JHDM1B) (31)<br>5A-D (JARID1A-D) (27-30)<br>NO66 (JMJD9) (25)
	H3K9me1 (tail)	Active TSSs (8)	**KMTs**<br>1C (G9a) (EHMT2) (32)<br>1D (GLP) (EHMT1) (33)<br>1E (SETDB1) (34)<br>8A (PRDM2) (35)<br>8E and F (PRDM3 and 16) (36)	**KDMs**<br>3A, B, C (JHDM2A, B, C) (37)<br>7B (JHDM1F or PHF8) (38)
	H3K27me1 (tail)	Intragenic regions of actively transcribed genes (39)	**KMTs**<br>6A and 6B (EZH2 and 1) (40,41)	**KDMs**<br>Unknown (42)
	H3K36me3 (tail)	Gene body and transcriptional elongation and splicing (43)	**KMTs**<br>3A (SETD2) (44)<br>8B (PRDM9) (45)	**KDMs**<br>4A and 4C (JHDM3A and C) (46,47)
	H3K79me1, 2, 3 (core)	Transcribed genomic region (48)	**KMTs**<br>4 (DOT1L) (48)	**KDMs**<br>2B (JHDM1B) (49)
	H3K4ac (tail)	Active promoters and TSSs (50)	**KATs**<br>2A (GCN5) (50)	**HDACs**<br>SIRT1 (SIR2 like protein 1) (50)
	H3K9ac (tail)	Active promoters and TSSs (51)	**KATs**<br>2A (GCN5) (52)<br>2B (PCAF) (52)	**HDACs**<br>2 (53) and 3 (54)<br>SIRT6 (SIR2 like protein 6) (55)
	H3K14ac (tail)	Active promoters (56)	**KATs**<br>2A (GCN5) (56)<br>7 (MYST2 or HBO1) (57)	**HDACs**<br>1 (58) and 3 (54)
	H3K18ac (tail)	Active TSSs (52)	**KATs**<br>3A (CBP) (52)<br>3B (P300) (52)	**HDACs**<br>SIRT2 (59)
	H3K23ac (tail)	Gene promoter regions (60)	**KATs**<br>6B (MORF) (60)	**HDACs**<br>6 (61)
	H3K27ac (tail)	Active promoters, TSSs and enhancers (1,62)	**KATs**<br>3A (CBP) (52)	**HDACs**<br>7 (63) and 8 (64)

			3B (P300) (52)	
	H3K56ac (core)	Active promoters (65)	**KATs** 3A (CBP) (66) 3B (P300) (66)	**HDACs** SIRT6 (67)
	H3K64ac (core)	Active promoters and TSSs (68)	**KATs** 3A (CBP) (68) 3B (P300) (68)	**HDACs** Unknown
	H3K122ac (core)	Active TSSs and enhancers (69) Induced by nuclear hormone receptor signaling, e.g. estrogen (69)	**KATs** 3A (CBP) (69) 3B (P300) (69)	**HDACs** Unknown
	H3R2me2a (tail)	Active promoters and enhancers (70)	**RMTs** PRMT6 (70)	**KDMs act as RDMs** 4E (JMJD2E) (71) 5C (JARID1C) (71) JMJD6 (72)
	H3R2me2s (tail)	Active promoters, TSSs and enhancers (poised genes) (73)	**RMTs** PRMT5 (73) PRMT7 (73)	**KDMs act as RDMs** 4E (JMJD2E) (71) 5C (JARID1C) (71) JMJD6 (72)
	H3R17me1, me2a (tail)	Active promoters (74)	**RMTs** PRMT4 (CARM1) (75)	**KDMs act as RDMs** Unknown
	H3R26me1, me2a (tail)	Promoter region of active genes (76)	**RMTs** PRMT4 (CARM1) (76)	**KDMs act as RDMs** 4E (JMJD2E) (71)
	H3R42me2 (core)	Unknown (77)	**RMTs** PRMT4 (CARM1) (78) PRMT6 (78)	**KDMs act as RDMs** Unknown
	H3S10P (tail)	Pericentric heterochromatin (79)	**Kinases** Aurora-B (79)	**Phosphatases** PP1 (80)
	H3Y41P (core)	Proximal promoters, TSSs and gene body (81)	**Kinases** JAK1 (81) and JAK2 (82)	**Phosphatases** Unknown
**Histone H4 (tail)**	H4K20me1	Gene body (83)	**KMTs** 5A (PR/SET07 or SETD8) (84)	**KDMs** 7B (JHDM1F or PHF8) (85)
	H4K5ac	Active TSSs and along gene bodies (86)	**KATs** 5 (TIP60) (87)	**HDACs** 3 (54)
	H4K8ac	Active promoters (88)	**KATs** 5 (TIP60) (87)	**HDACs** 3 (88)
	H4K12ac	Active promoters and TSSs of estrogen-induced genes (89)	**KATs** 5 (TIP60) (87)	**HDACs** 3 (54)
	H4K16ac	Promoter, TSSs, enhancers and gene body of active genes (90)	**KATs** 5 (TIP60) (87) 8 (MOF or MYST1) (91)	**HDACs** 1 and 2 (92) SIRT2 (93)
	H4R3me1, me2a	Active promoters (94)	**RMTs** PRMT1 (95)	**RDMs** JMJD6 (72)

Abbreviations: DNMT: DNA methyltransferase; TET: Ten-Eleven Translocation methylcytosine dioxygenase; TDG: thymine DNA glycosylase; TSS: transcriptional start site; K: lysine; KMT: histone lysine methyltransferase; KDM: histone lysine demethylase; me1: mono-methylated state; me2: di-methylated state; me3: tri-methylated state; ac: acetylation; KAT: histone lysine acetyltransferase; HDAC: histone deacetylase; CBP: CREB Binding Protein; ub1: monoubiquitination; RNF: Ring Finger Protein; USP44: Ubiquitin Specific Peptidase 44; MLL: Myeloid/Lymphoid Or Mixed-Lineage Leukemia; SETD: SET Domain Containing, Histone Lysine Methyltransferase; SMYD: SET and MYND Domain; PRDM: PR/SET Domain; LSD: Lysine Demethylase; JMJD: Jumonji Domain-Containing Protein; JARID: Jumonji, AT Rich Interactive Domain; JHDM: JmjC Domain-Containing Histone Demethylation Protein; GLP: G9a like protein; EHMT: Euchromatic Histone Lysine Methyltransferase; SETDB1: SET Domain Bifurcated Histone Lysine Methyltransferase 1; PHF8: PHD Finger Protein 8; EZH: Enhancer Of Zeste Polycomb Repressive Complex 2 (PRC2) Subunit; DOT1L: DOT1 Like Histone Lysine Methyltransferase; GCN5: General Control Of Amino Acid Synthesis Protein 5-Like 2; SIRT: NAD-dependent protein deacetylase

sirtuin; PCAF: P300/CBP-Associated Factor; TIP60: HIV-1 Tat Interactive Protein, 60kDa; P300: E1A-Binding Protein, 300kD; MORF: Monocytic Leukemia Zinc Finger Protein-Related Factor; R: arginine; me2a: asymmetric dimethylation; RMT: histone arginine methyltransferase; PRMT: protein arginine N-methyltransferase; RDM: histone N(ω)-methylarginine demethylases; CARM1: Coactivator Associated Arginine Methyltransferase 1; S: serine; P: phosphorylation; PP1: Protein Phosphatase 1; Y: tyrosine; JAK: Janus Kinase; MOF: Ortholog Of Drosophila Males Absent On The First.
The most common aliases and previous nomenclatures for some enzymes are in brackets.

REFERENCES

1. Roadmap Epigenomics, C., Kundaje, A., Meuleman, W., Ernst, J., Bilenky, M., Yen, A., Heravi-Moussavi, A., Kheradpour, P., Zhang, Z., Wang, J. *et al.* (2015) Integrative analysis of 111 reference human epigenomes. *Nature*, **518**, 317-330.
2. Liao, J., Karnik, R., Gu, H., Ziller, M.J., Clement, K., Tsankov, A.M., Akopian, V., Gifford, C.A., Donaghey, J., Galonska, C. *et al.* (2015) Targeted disruption of DNMT1, DNMT3A and DNMT3B in human embryonic stem cells. *Nat Genet*, **47**, 469-478.
3. Baubec, T., Colombo, D.F., Wirbelauer, C., Schmidt, J., Burger, L., Krebs, A.R., Akalin, A. and Schubeler, D. (2015) Genomic profiling of DNA methyltransferases reveals a role for DNMT3B in genic methylation. *Nature*, **520**, 243-247.
4. Chedin, F., Lieber, M.R. and Hsieh, C.L. (2002) The DNA methyltransferase-like protein DNMT3L stimulates de novo methylation by Dnmt3a. *Proc Natl Acad Sci U S A*, **99**, 16916-16921.
5. Tahiliani, M., Koh, K.P., Shen, Y., Pastor, W.A., Bandukwala, H., Brudno, Y., Agarwal, S., Iyer, L.M., Liu, D.R., Aravind, L. *et al.* (2009) Conversion of 5-methylcytosine to 5-hydroxymethylcytosine in mammalian DNA by MLL partner TET1. *Science*, **324**, 930-935.
6. Kohli, R.M. and Zhang, Y. (2013) TET enzymes, TDG and the dynamics of DNA demethylation. *Nature*, **502**, 472-479.
7. He, Y.F., Li, B.Z., Li, Z., Liu, P., Wang, Y., Tang, Q., Ding, J., Jia, Y., Chen, Z., Li, L. *et al.* (2011) Tet-mediated formation of 5-carboxylcytosine and its excision by TDG in mammalian DNA. *Science*, **333**, 1303-1307.
8. Barski, A., Cuddapah, S., Cui, K., Roh, T.Y., Schones, D.E., Wang, Z., Wei, G., Chepelev, I. and Zhao, K. (2007) High-resolution profiling of histone methylations in the human genome. *Cell*, **129**, 823-837.
9. Abell, A.N., Jordan, N.V., Huang, W., Prat, A., Midland, A.A., Johnson, N.L., Granger, D.A., Mieczkowski, P.A., Perou, C.M., Gomez, S.M. *et al.* (2011) MAP3K4/CBP-regulated H2B acetylation controls epithelial-mesenchymal transition in trophoblast stem cells. *Cell Stem Cell*, **8**, 525-537.

10. Mobley, R.J., Raghu, D., Duke, L.D., Abell-Hart, K., Zawistowski, J.S., Lutz, K., Gomez, S.M., Roy, S., Homayouni, R., Johnson, G.L. *et al.* (2017) MAP3K4 Controls the Chromatin Modifier HDAC6 during Trophoblast Stem Cell Epithelial-to-Mesenchymal Transition. *Cell Rep*, **18**, 2387-2400.
11. Kumar, V., Rayan, N.A., Muratani, M., Lim, S., Elanggovan, B., Xin, L., Lu, T., Makhija, H., Poschmann, J., Lufkin, T. *et al.* (2016) Comprehensive benchmarking reveals H2BK20 acetylation as a distinctive signature of cell-state-specific enhancers and promoters. *Genome Res*, **26**, 612-623.
12. Pavri, R., Zhu, B., Li, G., Trojer, P., Mandal, S., Shilatifard, A. and Reinberg, D. (2006) Histone H2B monoubiquitination functions cooperatively with FACT to regulate elongation by RNA polymerase II. *Cell*, **125**, 703-717.
13. Stegmeier, F., Rape, M., Draviam, V.M., Nalepa, G., Sowa, M.E., Ang, X.L., McDonald, E.R., 3rd, Li, M.Z., Hannon, G.J., Sorger, P.K. *et al.* (2007) Anaphase initiation is regulated by antagonistic ubiquitination and deubiquitination activities. *Nature*, **446**, 876-881.
14. Heintzman, N.D., Stuart, R.K., Hon, G., Fu, Y., Ching, C.W., Hawkins, R.D., Barrera, L.O., Van Calcar, S., Qu, C., Ching, K.A. *et al.* (2007) Distinct and predictive chromatin signatures of transcriptional promoters and enhancers in the human genome. *Nat Genet*, **39**, 311-318.
15. Dou, Y., Milne, T.A., Ruthenburg, A.J., Lee, S., Lee, J.W., Verdine, G.L., Allis, C.D. and Roeder, R.G. (2006) Regulation of MLL1 H3K4 methyltransferase activity by its core components. *Nat Struct Mol Biol*, **13**, 713-719.
16. Shinsky, S.A., Monteith, K.E., Viggiano, S. and Cosgrove, M.S. (2015) Biochemical reconstitution and phylogenetic comparison of human SET1 family core complexes involved in histone methylation. *J Biol Chem*, **290**, 6361-6375.
17. Lee, J.H. and Skalnik, D.G. (2008) Wdr82 is a C-terminal domain-binding protein that recruits the Setd1A Histone H3-Lys4 methyltransferase complex to transcription start sites of transcribed human genes. *Mol Cell Biol*, **28**, 609-618.
18. Lee, J.H. and Skalnik, D.G. (2005) CpG-binding protein (CXXC finger protein 1) is a component of the mammalian Set1 histone H3-Lys4 methyltransferase complex, the analogue of the yeast Set1/COMPASS complex. *J Biol Chem*, **280**, 41725-41731.
19. Abu-Farha, M., Lambert, J.P., Al-Madhoun, A.S., Elisma, F., Skerjanc, I.S. and Figeys, D. (2008) The tale of two domains: proteomics and genomics analysis of SMYD2, a new histone methyltransferase. *Mol Cell Proteomics*, **7**, 560-572.
20. Wilson, J.R., Jing, C., Walker, P.A., Martin, S.R., Howell, S.A., Blackburn, G.M., Gamblin, S.J. and Xiao, B. (2002) Crystal structure and functional analysis of the histone methyltransferase SET7/9. *Cell*, **111**, 105-115.
21. Wu, H., Mathioudakis, N., Diagouraga, B., Dong, A., Dombrovski, L., Baudat, F., Cusack, S., de Massy, B. and Kadlec, J. (2013) Molecular basis for the regulation of the H3K4 methyltransferase activity of PRDM9. *Cell Rep*, **5**, 13-20.

22. Shi, Y., Lan, F., Matson, C., Mulligan, P., Whetstine, J.R., Cole, P.A., Casero, R.A. and Shi, Y. (2004) Histone demethylation mediated by the nuclear amine oxidase homolog LSD1. *Cell*, **119**, 941-953.
23. Karytinos, A., Forneris, F., Profumo, A., Ciossani, G., Battaglioli, E., Binda, C. and Mattevi, A. (2009) A novel mammalian flavin-dependent histone demethylase. *J Biol Chem*, **284**, 17775-17782.
24. Xiang, Y., Zhu, Z., Han, G., Ye, X., Xu, B., Peng, Z., Ma, Y., Yu, Y., Lin, H., Chen, A.P. *et al.* (2007) JARID1B is a histone H3 lysine 4 demethylase up-regulated in prostate cancer. *Proc Natl Acad Sci U S A*, **104**, 19226-19231.
25. Sinha, K.M., Yasuda, H., Coombes, M.M., Dent, S.Y. and de Crombrugghe, B. (2010) Regulation of the osteoblast-specific transcription factor Osterix by NO66, a Jumonji family histone demethylase. *EMBO J*, **29**, 68-79.
26. Luo, X.G., Zhang, C.L., Zhao, W.W., Liu, Z.P., Liu, L., Mu, A., Guo, S., Wang, N., Zhou, H. and Zhang, T.C. (2014) Histone methyltransferase SMYD3 promotes MRTF-A-mediated transactivation of MYL9 and migration of MCF-7 breast cancer cells. *Cancer Lett*, **344**, 129-137.
27. Christensen, J., Agger, K., Cloos, P.A., Pasini, D., Rose, S., Sennels, L., Rappsilber, J., Hansen, K.H., Salcini, A.E. and Helin, K. (2007) RBP2 belongs to a family of demethylases, specific for tri-and dimethylated lysine 4 on histone 3. *Cell*, **128**, 1063-1076.
28. Yamane, K., Tateishi, K., Klose, R.J., Fang, J., Fabrizio, L.A., Erdjument-Bromage, H., Taylor-Papadimitriou, J., Tempst, P. and Zhang, Y. (2007) PLU-1 is an H3K4 demethylase involved in transcriptional repression and breast cancer cell proliferation. *Mol Cell*, **25**, 801-812.
29. Tahiliani, M., Mei, P., Fang, R., Leonor, T., Rutenberg, M., Shimizu, F., Li, J., Rao, A. and Shi, Y. (2007) The histone H3K4 demethylase SMCX links REST target genes to X-linked mental retardation. *Nature*, **447**, 601-605.
30. Lee, M.G., Norman, J., Shilatifard, A. and Shiekhattar, R. (2007) Physical and functional association of a trimethyl H3K4 demethylase and Ring6a/MBLR, a polycomb-like protein. *Cell*, **128**, 877-887.
31. Frescas, D., Guardavaccaro, D., Bassermann, F., Koyama-Nasu, R. and Pagano, M. (2007) JHDM1B/FBXL10 is a nucleolar protein that represses transcription of ribosomal RNA genes. *Nature*, **450**, 309-313.
32. Tachibana, M., Sugimoto, K., Fukushima, T. and Shinkai, Y. (2001) Set domain-containing protein, G9a, is a novel lysine-preferring mammalian histone methyltransferase with hyperactivity and specific selectivity to lysines 9 and 27 of histone H3. *J Biol Chem*, **276**, 25309-25317.
33. Ogawa, H., Ishiguro, K., Gaubatz, S., Livingston, D.M. and Nakatani, Y. (2002) A complex with chromatin modifiers that occupies E2F- and Myc-responsive genes in G0 cells. *Science*, **296**, 1132-1136.

34. Schultz, D.C., Ayyanathan, K., Negorev, D., Maul, G.G. and Rauscher, F.J., 3rd. (2002) SETDB1: a novel KAP-1-associated histone H3, lysine 9-specific methyltransferase that contributes to HP1-mediated silencing of euchromatic genes by KRAB zinc-finger proteins. *Genes Dev*, **16**, 919-932.
35. Kim, K.C., Geng, L. and Huang, S. (2003) Inactivation of a histone methyltransferase by mutations in human cancers. *Cancer Res*, **63**, 7619-7623.
36. Pinheiro, I., Margueron, R., Shukeir, N., Eisold, M., Fritzsch, C., Richter, F.M., Mittler, G., Genoud, C., Goyama, S., Kurokawa, M. *et al.* (2012) Prdm3 and Prdm16 are H3K9me1 methyltransferases required for mammalian heterochromatin integrity. *Cell*, **150**, 948-960.
37. Yamane, K., Toumazou, C., Tsukada, Y., Erdjument-Bromage, H., Tempst, P., Wong, J. and Zhang, Y. (2006) JHDM2A, a JmjC-containing H3K9 demethylase, facilitates transcription activation by androgen receptor. *Cell*, **125**, 483-495.
38. Loenarz, C., Ge, W., Coleman, M.L., Rose, N.R., Cooper, C.D., Klose, R.J., Ratcliffe, P.J. and Schofield, C.J. (2010) PHF8, a gene associated with cleft lip/palate and mental retardation, encodes for an Nepsilon-dimethyl lysine demethylase. *Hum Mol Genet*, **19**, 217-222.
39. Ferrari, K.J., Scelfo, A., Jammula, S., Cuomo, A., Barozzi, I., Stutzer, A., Fischle, W., Bonaldi, T. and Pasini, D. (2014) Polycomb-dependent H3K27me1 and H3K27me2 regulate active transcription and enhancer fidelity. *Mol Cell*, **53**, 49-62.
40. Shen, X., Liu, Y., Hsu, Y.J., Fujiwara, Y., Kim, J., Mao, X., Yuan, G.C. and Orkin, S.H. (2008) EZH1 mediates methylation on histone H3 lysine 27 and complements EZH2 in maintaining stem cell identity and executing pluripotency. *Mol Cell*, **32**, 491-502.
41. Kuzmichev, A., Nishioka, K., Erdjument-Bromage, H., Tempst, P. and Reinberg, D. (2002) Histone methyltransferase activity associated with a human multiprotein complex containing the Enhancer of Zeste protein. *Genes Dev*, **16**, 2893-2905.
42. Swigut, T. and Wysocka, J. (2007) H3K27 demethylases, at long last. *Cell*, **131**, 29-32.
43. Wagner, E.J. and Carpenter, P.B. (2012) Understanding the language of Lys36 methylation at histone H3. *Nat Rev Mol Cell Biol*, **13**, 115-126.
44. Edmunds, J.W., Mahadevan, L.C. and Clayton, A.L. (2008) Dynamic histone H3 methylation during gene induction: HYPB/Setd2 mediates all H3K36 trimethylation. *EMBO J*, **27**, 406-420.
45. Eram, M.S., Bustos, S.P., Lima-Fernandes, E., Siarheyeva, A., Senisterra, G., Hajian, T., Chau, I., Duan, S., Wu, H., Dombrovski, L. *et al.* (2014) Trimethylation of histone H3 lysine 36 by human methyltransferase PRDM9 protein. *J Biol Chem*, **289**, 12177-12188.

46. Klose, R.J., Yamane, K., Bae, Y., Zhang, D., Erdjument-Bromage, H., Tempst, P., Wong, J. and Zhang, Y. (2006) The transcriptional repressor JHDM3A demethylates trimethyl histone H3 lysine 9 and lysine 36. *Nature*, **442**, 312-316.
47. Cloos, P.A., Christensen, J., Agger, K., Maiolica, A., Rappsilber, J., Antal, T., Hansen, K.H. and Helin, K. (2006) The putative oncogene GASC1 demethylates tri- and dimethylated lysine 9 on histone H3. *Nature*, **442**, 307-311.
48. Steger, D.J., Lefterova, M.I., Ying, L., Stonestrom, A.J., Schupp, M., Zhuo, D., Vakoc, A.L., Kim, J.E., Chen, J., Lazar, M.A. *et al.* (2008) DOT1L/KMT4 recruitment and H3K79 methylation are ubiquitously coupled with gene transcription in mammalian cells. *Mol Cell Biol*, **28**, 2825-2839.
49. Kang, J.Y., Kim, J.Y., Kim, K.B., Park, J.W., Cho, H., Hahm, J.Y., Chae, Y.C., Kim, D., Kook, H., Rhee, S. *et al.* (2018) KDM2B is a histone H3K79 demethylase and induces transcriptional repression via sirtuin-1-mediated chromatin silencing. *FASEB J*, **32**, 5737-5750.
50. Guillemette, B., Drogaris, P., Lin, H.H., Armstrong, H., Hiragami-Hamada, K., Imhof, A., Bonneil, E., Thibault, P., Verreault, A. and Festenstein, R.J. (2011) H3 lysine 4 is acetylated at active gene promoters and is regulated by H3 lysine 4 methylation. *PLoS Genet*, **7**, e1001354.
51. Bernstein, B.E., Kamal, M., Lindblad-Toh, K., Bekiranov, S., Bailey, D.K., Huebert, D.J., McMahon, S., Karlsson, E.K., Kulbokas, E.J., 3rd, Gingeras, T.R. *et al.* (2005) Genomic maps and comparative analysis of histone modifications in human and mouse. *Cell*, **120**, 169-181.
52. Jin, Q., Yu, L.R., Wang, L., Zhang, Z., Kasper, L.H., Lee, J.E., Wang, C., Brindle, P.K., Dent, S.Y. and Ge, K. (2011) Distinct roles of GCN5/PCAF-mediated H3K9ac and CBP/p300-mediated H3K18/27ac in nuclear receptor transactivation. *EMBO J*, **30**, 249-262.
53. Somanath, P., Herndon Klein, R. and Knoepfler, P.S. (2017) CRISPR-mediated HDAC2 disruption identifies two distinct classes of target genes in human cells. *PLoS One*, **12**, e0185627.
54. Bhaskara, S., Knutson, S.K., Jiang, G., Chandrasekharan, M.B., Wilson, A.J., Zheng, S., Yenamandra, A., Locke, K., Yuan, J.L., Bonine-Summers, A.R. *et al.* (2010) Hdac3 is essential for the maintenance of chromatin structure and genome stability. *Cancer Cell*, **18**, 436-447.
55. Michishita, E., McCord, R.A., Berber, E., Kioi, M., Padilla-Nash, H., Damian, M., Cheung, P., Kusumoto, R., Kawahara, T.L., Barrett, J.C. *et al.* (2008) SIRT6 is a histone H3 lysine 9 deacetylase that modulates telomeric chromatin. *Nature*, **452**, 492-496.
56. Johnsson, A., Durand-Dubief, M., Xue-Franzen, Y., Ronnerblad, M., Ekwall, K. and Wright, A. (2009) HAT-HDAC interplay modulates global histone H3K14 acetylation in gene-coding regions during stress. *EMBO Rep*, **10**, 1009-1014.

57. Kueh, A.J., Dixon, M.P., Voss, A.K. and Thomas, T. (2011) HBO1 is required for H3K14 acetylation and normal transcriptional activity during embryonic development. *Mol Cell Biol*, **31**, 845-860.
58. Wu, M., Hayward, D., Kalin, J.H., Song, Y., Schwabe, J.W. and Cole, P.A. (2018) Lysine-14 acetylation of histone H3 in chromatin confers resistance to the deacetylase and demethylase activities of an epigenetic silencing complex. *Elife*, **7**.
59. Damodaran, S., Damaschke, N., Gawdzik, J., Yang, B., Shi, C., Allen, G.O., Huang, W., Denu, J. and Jarrard, D. (2017) Dysregulation of Sirtuin 2 (SIRT2) and histone H3K18 acetylation pathways associates with adverse prostate cancer outcomes. *BMC Cancer*, **17**, 874.
60. Klein, B.J., Jang, S.M., Lachance, C., Mi, W., Lyu, J., Sakuraba, S., Krajewski, K., Wang, W.W., Sidoli, S., Liu, J. *et al.* (2019) Histone H3K23-specific acetylation by MORF is coupled to H3K14 acylation. *Nat Commun*, **10**, 4724.
61. Guo, X., Fang, Z.M., Wei, X., Huo, B., Yi, X., Cheng, C., Chen, J., Zhu, X.H., Bokha, A. and Jiang, D.S. (2019) HDAC6 is associated with the formation of aortic dissection in human. *Mol Med*, **25**, 10.
62. Rada-Iglesias, A., Bajpai, R., Swigut, T., Brugmann, S.A., Flynn, R.A. and Wysocka, J. (2011) A unique chromatin signature uncovers early developmental enhancers in humans. *Nature*, **470**, 279-283.
63. Caslini, C., Hong, S., Ban, Y.J., Chen, X.S. and Ince, T.A. (2019) HDAC7 regulates histone 3 lysine 27 acetylation and transcriptional activity at super-enhancer-associated genes in breast cancer stem cells. *Oncogene*, **38**, 6599-6614.
64. Chen, L.F., Lin, Y.T., Gallegos, D.A., Hazlett, M.F., Gomez-Schiavon, M., Yang, M.G., Kalmeta, B., Zhou, A.S., Holtzman, L., Gersbach, C.A. *et al.* (2019) Enhancer Histone Acetylation Modulates Transcriptional Bursting Dynamics of Neuronal Activity-Inducible Genes. *Cell Rep*, **26**, 1174-1188 e1175.
65. Williams, S.K., Truong, D. and Tyler, J.K. (2008) Acetylation in the globular core of histone H3 on lysine-56 promotes chromatin disassembly during transcriptional activation. *Proc Natl Acad Sci U S A*, **105**, 9000-9005.
66. Das, C., Lucia, M.S., Hansen, K.C. and Tyler, J.K. (2009) CBP/p300-mediated acetylation of histone H3 on lysine 56. *Nature*, **459**, 113-117.
67. Michishita, E., McCord, R.A., Boxer, L.D., Barber, M.F., Hong, T., Gozani, O. and Chua, K.F. (2009) Cell cycle-dependent deacetylation of telomeric histone H3 lysine K56 by human SIRT6. *Cell Cycle*, **8**, 2664-2666.
68. Di Cerbo, V., Mohn, F., Ryan, D.P., Montellier, E., Kacem, S., Tropberger, P., Kallis, E., Holzner, M., Hoerner, L., Feldmann, A. *et al.* (2014) Acetylation of histone H3 at lysine 64 regulates nucleosome dynamics and facilitates transcription. *Elife*, **3**, e01632.
69. Tropberger, P., Pott, S., Keller, C., Kamieniarz-Gdula, K., Caron, M., Richter, F., Li, G., Mittler, G., Liu, E.T., Buhler, M. *et al.* (2013) Regulation of transcription through

acetylation of H3K122 on the lateral surface of the histone octamer. *Cell*, **152**, 859-872.

70. Bouchard, C., Sahu, P., Meixner, M., Notzold, R.R., Rust, M.B., Kremmer, E., Feederle, R., Hart-Smith, G., Finkernagel, F., Bartkuhn, M. *et al.* (2018) Genomic Location of PRMT6-Dependent H3R2 Methylation Is Linked to the Transcriptional Outcome of Associated Genes. *Cell Rep*, **24**, 3339-3352.
71. Walport, L.J., Hopkinson, R.J., Chowdhury, R., Schiller, R., Ge, W., Kawamura, A. and Schofield, C.J. (2016) Arginine demethylation is catalysed by a subset of JmjC histone lysine demethylases. *Nat Commun*, **7**, 11974.
72. Chang, B., Chen, Y., Zhao, Y. and Bruick, R.K. (2007) JMJD6 is a histone arginine demethylase. *Science*, **318**, 444-447.
73. Migliori, V., Muller, J., Phalke, S., Low, D., Bezzi, M., Mok, W.C., Sahu, S.K., Gunaratne, J., Capasso, P., Bassi, C. *et al.* (2012) Symmetric dimethylation of H3R2 is a newly identified histone mark that supports euchromatin maintenance. *Nat Struct Mol Biol*, **19**, 136-144.
74. Bauer, U.M., Daujat, S., Nielsen, S.J., Nightingale, K. and Kouzarides, T. (2002) Methylation at arginine 17 of histone H3 is linked to gene activation. *EMBO Rep*, **3**, 39-44.
75. Selvi, B.R., Batta, K., Kishore, A.H., Mantelingu, K., Varier, R.A., Balasubramanyam, K., Pradhan, S.K., Dasgupta, D., Sriram, S., Agrawal, S. *et al.* (2010) Identification of a novel inhibitor of coactivator-associated arginine methyltransferase 1 (CARM1)-mediated methylation of histone H3 Arg-17. *J Biol Chem*, **285**, 7143-7152.
76. Blanc, R.S. and Richard, S. (2017) Arginine Methylation: The Coming of Age. *Mol Cell*, **65**, 8-24.
77. Kebede, A.F., Schneider, R. and Daujat, S. (2015) Novel types and sites of histone modifications emerge as players in the transcriptional regulation contest. *FEBS J*, **282**, 1658-1674.
78. Casadio, F., Lu, X., Pollock, S.B., LeRoy, G., Garcia, B.A., Muir, T.W., Roeder, R.G. and Allis, C.D. (2013) H3R42me2a is a histone modification with positive transcriptional effects. *Proc Natl Acad Sci U S A*, **110**, 14894-14899.
79. Hirota, T., Lipp, J.J., Toh, B.H. and Peters, J.M. (2005) Histone H3 serine 10 phosphorylation by Aurora B causes HP1 dissociation from heterochromatin. *Nature*, **438**, 1176-1180.
80. Adhvaryu, K.K. and Selker, E.U. (2008) Protein phosphatase PP1 is required for normal DNA methylation in Neurospora. *Genes Dev*, **22**, 3391-3396.
81. Rui, L., Drennan, A.C., Ceribelli, M., Zhu, F., Wright, G.W., Huang, D.W., Xiao, W., Li, Y., Grindle, K.M., Lu, L. *et al.* (2016) Epigenetic gene regulation by Janus kinase 1 in diffuse large B-cell lymphoma. *Proc Natl Acad Sci U S A*, **113**, E7260-E7267.

82. Dawson, M.A., Bannister, A.J., Gottgens, B., Foster, S.D., Bartke, T., Green, A.R. and Kouzarides, T. (2009) JAK2 phosphorylates histone H3Y41 and excludes HP1alpha from chromatin. *Nature*, **461**, 819-822.
83. Beck, D.B., Oda, H., Shen, S.S. and Reinberg, D. (2012) PR-Set7 and H4K20me1: at the crossroads of genome integrity, cell cycle, chromosome condensation, and transcription. *Genes Dev*, **26**, 325-337.
84. Fang, J., Feng, Q., Ketel, C.S., Wang, H., Cao, R., Xia, L., Erdjument-Bromage, H., Tempst, P., Simon, J.A. and Zhang, Y. (2002) Purification and functional characterization of SET8, a nucleosomal histone H4-lysine 20-specific methyltransferase. *Curr Biol*, **12**, 1086-1099.
85. Liu, W., Tanasa, B., Tyurina, O.V., Zhou, T.Y., Gassmann, R., Liu, W.T., Ohgi, K.A., Benner, C., Garcia-Bassets, I., Aggarwal, A.K. *et al.* (2010) PHF8 mediates histone H4 lysine 20 demethylation events involved in cell cycle progression. *Nature*, **466**, 508-512.
86. Wang, Z., Zang, C., Rosenfeld, J.A., Schones, D.E., Barski, A., Cuddapah, S., Cui, K., Roh, T.Y., Peng, W., Zhang, M.Q. *et al.* (2008) Combinatorial patterns of histone acetylations and methylations in the human genome. *Nat Genet*, **40**, 897-903.
87. Utley, R.T. and Cote, J. (2003) The MYST family of histone acetyltransferases. *Curr Top Microbiol Immunol*, **274**, 203-236.
88. McQuown, S.C., Barrett, R.M., Matheos, D.P., Post, R.J., Rogge, G.A., Alenghat, T., Mullican, S.E., Jones, S., Rusche, J.R., Lazar, M.A. *et al.* (2011) HDAC3 is a critical negative regulator of long-term memory formation. *J Neurosci*, **31**, 764-774.
89. Nagarajan, S., Benito, E., Fischer, A. and Johnsen, S.A. (2015) H4K12ac is regulated by estrogen receptor-alpha and is associated with BRD4 function and inducible transcription. *Oncotarget*, **6**, 7305-7317.
90. Taylor, G.C., Eskeland, R., Hekimoglu-Balkan, B., Pradeepa, M.M. and Bickmore, W.A. (2013) H4K16 acetylation marks active genes and enhancers of embryonic stem cells, but does not alter chromatin compaction. *Genome Res*, **23**, 2053-2065.
91. Taipale, M., Rea, S., Richter, K., Vilar, A., Lichter, P., Imhof, A. and Akhtar, A. (2005) hMOF histone acetyltransferase is required for histone H4 lysine 16 acetylation in mammalian cells. *Mol Cell Biol*, **25**, 6798-6810.
92. Bhaskara, S., Jacques, V., Rusche, J.R., Olson, E.N., Cairns, B.R. and Chandrasekharan, M.B. (2013) Histone deacetylases 1 and 2 maintain S-phase chromatin and DNA replication fork progression. *Epigenetics Chromatin*, **6**, 27.
93. Vaquero, A., Scher, M.B., Lee, D.H., Sutton, A., Cheng, H.L., Alt, F.W., Serrano, L., Sternglanz, R. and Reinberg, D. (2006) SirT2 is a histone deacetylase with preference for histone H4 Lys 16 during mitosis. *Genes Dev*, **20**, 1256-1261.
94. Bedford, M.T. and Richard, S. (2005) Arginine methylation an emerging regulator of protein function. *Mol Cell*, **18**, 263-272.

95. Wang, H., Huang, Z.Q., Xia, L., Feng, Q., Erdjument-Bromage, H., Strahl, B.D., Briggs, S.D., Allis, C.D., Wong, J., Tempst, P. *et al.* (2001) Methylation of histone H4 at arginine 3 facilitating transcriptional activation by nuclear hormone receptor. *Science*, **293**, 853-857.
