# Supplementary material for: Epigenome engineering: new technologies for precision medicine
Source: Nucleic Acids Res. 2020 Nov 16;48(22):12453–82. doi: 10.1093/nar/gkaa1000 (PMC7736826; doi:10.1093/nar/gkaa1000)
Supplement: gkaa1000_Supplemental_Files [file gkaa1000_supplemental_files.zip › Supplementary Table S2.pdf]

Supplementary Table S2. Chromatin modifications, writers and erasers of epigenetic marks for gene transcriptional repression

Chromatin	Modifications for gene transcriptional REPRESSION	Genomic Location	Epigenetic Enzymes (Writers)	Epigenetic Enzymes (Erasers)
**DNA**	High CpG methylation	Inactive promoters, enhancers and within transcribed gene bodies (1,2)	**DNMTs**<br>1 (maintenance) (3)<br>3A and B (*de novo*) (2,3)<br>3L (cofactor) (4)	**DNA demethylases**<br>TET1, TET2, TET3 (5,6)<br>TDG (7)
**Histone H2A (tail)**	H2AK119uq	Promoter regions (8)	**Ubiquitin E3 ligases**<br>RNF2 (RING1B) (8)	**Deubiquitinating hydrolases (DUBS)**<br>USP3 (9)
**Histone H3 (core and tail)**	H3K9me2 (tail)	Characteristic mark of the inactive X chromosome (Xi) (10)	**KMTs**<br>1A and 1B (SUV39H1 and 2) (11)<br>1C (G9a or EHMT2) (12)<br>1D (GLP or EHMT1) (13)<br>1E (SETDB1) (14)<br>8A (PRDM2) (15)<br>8D (PRDM8) (16)	**KDMs**<br>3A-C (JHDM2A-C) (17)<br>4A-D (JHDM3A-D) (18-21)<br>7A (JHDM1D or KIAA1718) (22)<br>7B (JHDM1F or PHF8) (23)
	H3K9me3 (tail)	Associated with constitutive heterochromatin (24)	**KMTs**<br>1A and 1B (SUV39H1 and 2) (11)<br>1E and 1F (SETDB1 and 2) (25,26)<br>8A (PRDM2) (15)	**KDMs**<br>4A-D (JHDM3A-D) (18-21)
	H3K27me2 (tail)	Large chromatin domains and protective function by preventing firing of non-specific enhancers (27)	**KMTs**<br>6A and 6B (EZH2 and 1) (28,29)	**KDMs**<br>6A (UTX) (30)<br>6B (JMJD3) (31)<br>6C (UTY) (32)<br>7A (JHDM1D or KIAA1718) (22)<br>7B (JHDM1F or PHF8) (23)
	H3K27me3 (tail)	Facultative inactive or bivalent TSSs and enhancers (poised genes), and some gene bodies (33); and Xi (34)	**KMTs**<br>6A and 6B (EZH2 and 1) (28,29)	**KDMs**<br>6A (UTX) (30)<br>6B (JMJD3) (31)<br>6C (UTY) (32)
	H3K36me1 (tail)	Gene bodies (35)	**KMTs**<br>3A (SETD2) (36,37)<br>3B, 3F or 3G (NSD1 and 2) (38,39)<br>8B (PRDM9) (40)	**KDMs**<br>2A and 2B (JHDM1A and B) (41,42)
	H3K36me2 (tail)	Gene body and intergenic regions; Inhibits cryptic intragenic transcription; Near DNA double-strand breaks (35,43-45)	**KMTs**<br>2H (ASH1L) (46)<br>3B, F, G (NSD1, 2, 3) (38,39,47)<br>3C (SMYD2) (48)<br>8B (PRDM9) (40)<br>SETD3 (49)<br>SETMAR (45,50)	**KDMs**<br>2A and 2B (JHDM1A and B) (41,42)
	H3K56me3 (core)	Pericentromeric and heterochromatic foci (51)	**KMTs**<br>1A and 1B (SUV39H1 and 2) (51)	**KDMs**<br>4A-E (JMJD2A-E) (51)
	H3K64me3 (core)	Pericentromeric, subtelomeric repeats and heterochromatin foci (52,53)	**KMTs**<br>1A and 1B (SUV39H1 and 2) (52)	**KDMs**<br>4C (JMJD2C) (53)
	H3R2me2a (tail)	Promoter regions and evenly distributed on chromosome arms (54,55)	**RMTs**<br>PRMT6 (56)	**KDMs act as RDMs**<br>4E (JMJD2E) (57)<br>5C (JARID1C) (57)<br>JMJD6 (58)
	H3R8me2a (tail)	Promoter regions (poised genes) (59)	**RMTs**<br>PRMT2 (59)	**KDMs act as RDMs**<br>4E (JMJD2E) (57)<br>5C (JARID1C) (57)
	H3R8me2s (tail)	Promoter regions (60)	**RMTs**<br>PRMT5 (61)	**KDMs act as RDMs**<br>4E (JMJD2E) (57)<br>5C (JARID1C) (57)

**Histone H4 (tail)**	H4K20me2	Damaged DNA, DNA replication origins (62,63)	**KMTs**<br>5B and 5C<br>(SUV4-20H1 and H2) (64-66)	**KDMs**<br>1A (LSD1n) (67)<br>9 (RSBN1) (68)
	H4K20me3	Promoters, bivalent TSSs and gene bodies (poised genes), and repetitive elements (33,69)	**KMTs**<br>5B and 5C<br>(SUV4-20H1 and H2) (64-66)	**KDMs**<br>7C (PHF2) (70)
	H4R3me2s	Promoter and gene body regions (71)	**RMTs**<br>PRMT5 (72)	**KDMs act as RDMs**<br>3B (JMJD1B) (71)<br>4E (JMJD2E) (57)

Abbreviations: DNMT: DNA methyltransferase; TET: Ten-Eleven Translocation methylcytosine dioxygenase; TDG: thymine DNA glycosylase; K: lysine; uq: ubiquitination; RNF2: Ring Finger Protein 2; USP3: Ubiquitin Specific Peptidase 3; me1: mono-methylated state; me2: di-methylated state; me3: tri-methylated state; KMT: histone lysine methyltransferase; KDM: histone lysine demethylase; SUV39H: Suppressor Of Variegation 3-9 Homolog; EHMT: Euchromatic Histone Lysine Methyltransferase ; SETDB1: SET Domain Bifurcated Histone Lysine Methyltransferase 1; PRDM: PR/SET Domain; JHDM: JmjC Domain-Containing Histone Demethylation Protein; PHF: PHD Finger Protein; Xi: inactive X chromosome; EZH: Enhancer Of Zeste Polycomb Repressive Complex 2 (PRC2) Subunit; UTX: Ubiquitously-Transcribed X Chromosome Tetratricopeptide Repeat Protein; JMJD: Jumonji Domain-Containing Protein; UTY: Ubiquitously Transcribed Tetratricopeptide Repeat Containing, Y-Linked; TSS: transcriptional start site; SETD: SET Domain Containing, Histone Lysine Methyltransferase; NSD: Nuclear Receptor Binding SET Domain Protein; ASH1L: ASH1 Like Histone Lysine Methyltransferase; SMYD2: SET And MYND Domain Containing 2; SETMAR: SET Domain And Mariner Transposase Fusion Gene; R: arginine; me2a: asymmetric di-methylated state; me2s: symmetric di-methylated state; RMT: histone arginine methyltransferase; PRMT: protein arginine N-methyltransferase; RDM: histone N(ω)-methylarginine demethylases; JARID: Jumonji, AT Rich Interactive Domain; SUV4-20H: Suppressor Of Variegation 4-20 Homolog; LSD1n: Lysine Demethylase 1A, neuron-specific isoform; RSBN1: Round Spermatid Basic Protein 1.
The most common aliases and previous nomenclatures for some enzymes are in brackets.

REFERENCES

1. Roadmap Epigenomics, C., Kundaje, A., Meuleman, W., Ernst, J., Bilenky, M., Yen, A., Heravi-Moussavi, A., Kheradpour, P., Zhang, Z., Wang, J. *et al.* (2015) Integrative analysis of 111 reference human epigenomes. *Nature*, **518**, 317-330.
2. Baubec, T., Colombo, D.F., Wirbelauer, C., Schmidt, J., Burger, L., Krebs, A.R., Akalin, A. and Schubeler, D. (2015) Genomic profiling of DNA methyltransferases reveals a role for DNMT3B in genic methylation. *Nature*, **520**, 243-247.
3. Liao, J., Karnik, R., Gu, H., Ziller, M.J., Clement, K., Tsankov, A.M., Akopian, V., Gifford, C.A., Donaghey, J., Galonska, C. *et al.* (2015) Targeted disruption of

DNMT1, DNMT3A and DNMT3B in human embryonic stem cells. *Nat Genet*, **47**, 469-478.

4. Chedin, F., Lieber, M.R. and Hsieh, C.L. (2002) The DNA methyltransferase-like protein DNMT3L stimulates de novo methylation by Dnmt3a. *Proc Natl Acad Sci U S A*, **99**, 16916-16921.
5. Tahiliani, M., Koh, K.P., Shen, Y., Pastor, W.A., Bandukwala, H., Brudno, Y., Agarwal, S., Iyer, L.M., Liu, D.R., Aravind, L. *et al.* (2009) Conversion of 5-methylcytosine to 5-hydroxymethylcytosine in mammalian DNA by MLL partner TET1. *Science*, **324**, 930-935.
6. Kohli, R.M. and Zhang, Y. (2013) TET enzymes, TDG and the dynamics of DNA demethylation. *Nature*, **502**, 472-479.
7. He, Y.F., Li, B.Z., Li, Z., Liu, P., Wang, Y., Tang, Q., Ding, J., Jia, Y., Chen, Z., Li, L. *et al.* (2011) Tet-mediated formation of 5-carboxylcytosine and its excision by TDG in mammalian DNA. *Science*, **333**, 1303-1307.
8. Wang, H., Wang, L., Erdjument-Bromage, H., Vidal, M., Tempst, P., Jones, R.S. and Zhang, Y. (2004) Role of histone H2A ubiquitination in Polycomb silencing. *Nature*, **431**, 873-878.
9. Nicassio, F., Corrado, N., Vissers, J.H., Areces, L.B., Bergink, S., Marteijn, J.A., Geverts, B., Houtsmuller, A.B., Vermeulen, W., Di Fiore, P.P. *et al.* (2007) Human USP3 is a chromatin modifier required for S phase progression and genome stability. *Curr Biol*, **17**, 1972-1977.
10. Rougeulle, C., Chaumeil, J., Sarma, K., Allis, C.D., Reinberg, D., Avner, P. and Heard, E. (2004) Differential histone H3 Lys-9 and Lys-27 methylation profiles on the X chromosome. *Mol Cell Biol*, **24**, 5475-5484.
11. Rea, S., Eisenhaber, F., O'Carroll, D., Strahl, B.D., Sun, Z.W., Schmid, M., Opravil, S., Mechtler, K., Ponting, C.P., Allis, C.D. *et al.* (2000) Regulation of chromatin structure by site-specific histone H3 methyltransferases. *Nature*, **406**, 593-599.
12. Tachibana, M., Sugimoto, K., Nozaki, M., Ueda, J., Ohta, T., Ohki, M., Fukuda, M., Takeda, N., Niida, H., Kato, H. *et al.* (2002) G9a histone methyltransferase plays a dominant role in euchromatic histone H3 lysine 9 methylation and is essential for early embryogenesis. *Genes Dev*, **16**, 1779-1791.
13. Tachibana, M., Ueda, J., Fukuda, M., Takeda, N., Ohta, T., Iwanari, H., Sakihama, T., Kodama, T., Hamakubo, T. and Shinkai, Y. (2005) Histone methyltransferases G9a and GLP form heteromeric complexes and are both crucial for methylation of euchromatin at H3-K9. *Genes Dev*, **19**, 815-826.
14. Schultz, D.C., Ayyanathan, K., Negorev, D., Maul, G.G. and Rauscher, F.J., 3rd. (2002) SETDB1: a novel KAP-1-associated histone H3, lysine 9-specific methyltransferase that contributes to HP1-mediated silencing of euchromatic genes by KRAB zinc-finger proteins. *Genes Dev*, **16**, 919-932.

15. Kim, K.C., Geng, L. and Huang, S. (2003) Inactivation of a histone methyltransferase by mutations in human cancers. *Cancer Res*, **63**, 7619-7623.
16. Eom, G.H., Kim, K., Kim, S.M., Kee, H.J., Kim, J.Y., Jin, H.M., Kim, J.R., Kim, J.H., Choe, N., Kim, K.B. *et al.* (2009) Histone methyltransferase PRDM8 regulates mouse testis steroidogenesis. *Biochem Biophys Res Commun*, **388**, 131-136.
17. Yamane, K., Toumazou, C., Tsukada, Y., Erdjument-Bromage, H., Tempst, P., Wong, J. and Zhang, Y. (2006) JHDM2A, a JmjC-containing H3K9 demethylase, facilitates transcription activation by androgen receptor. *Cell*, **125**, 483-495.
18. Fodor, B.D., Kubicek, S., Yonezawa, M., O'Sullivan, R.J., Sengupta, R., Perez-Burgos, L., Opravil, S., Mechtler, K., Schotta, G. and Jenuwein, T. (2006) Jmjd2b antagonizes H3K9 trimethylation at pericentric heterochromatin in mammalian cells. *Genes Dev*, **20**, 1557-1562.
19. Klose, R.J., Yamane, K., Bae, Y., Zhang, D., Erdjument-Bromage, H., Tempst, P., Wong, J. and Zhang, Y. (2006) The transcriptional repressor JHDM3A demethylates trimethyl histone H3 lysine 9 and lysine 36. *Nature*, **442**, 312-316.
20. Cloos, P.A., Christensen, J., Agger, K., Maiolica, A., Rappsilber, J., Antal, T., Hansen, K.H. and Helin, K. (2006) The putative oncogene GASC1 demethylates tri- and dimethylated lysine 9 on histone H3. *Nature*, **442**, 307-311.
21. Whetstine, J.R., Nottke, A., Lan, F., Huarte, M., Smolikov, S., Chen, Z., Spooner, E., Li, E., Zhang, G., Colaiacovo, M. *et al.* (2006) Reversal of histone lysine trimethylation by the JMJD2 family of histone demethylases. *Cell*, **125**, 467-481.
22. Tsukada, Y., Ishitani, T. and Nakayama, K.I. (2010) KDM7 is a dual demethylase for histone H3 Lys 9 and Lys 27 and functions in brain development. *Genes Dev*, **24**, 432-437.
23. Loenarz, C., Ge, W., Coleman, M.L., Rose, N.R., Cooper, C.D., Klose, R.J., Ratcliffe, P.J. and Schofield, C.J. (2010) PHF8, a gene associated with cleft lip/palate and mental retardation, encodes for an Nepsilon-dimethyl lysine demethylase. *Hum Mol Genet*, **19**, 217-222.
24. Becker, J.S., Nicetto, D. and Zaret, K.S. (2016) H3K9me3-Dependent Heterochromatin: Barrier to Cell Fate Changes. *Trends Genet*, **32**, 29-41.
25. Ayyanathan, K., Lechner, M.S., Bell, P., Maul, G.G., Schultz, D.C., Yamada, Y., Tanaka, K., Torigoe, K. and Rauscher, F.J., 3rd. (2003) Regulated recruitment of HP1 to a euchromatic gene induces mitotically heritable, epigenetic gene silencing: a mammalian cell culture model of gene variegation. *Genes Dev*, **17**, 1855-1869.
26. Falandry, C., Fourel, G., Galy, V., Ristriani, T., Horard, B., Bensimon, E., Salles, G., Gilson, E. and Magdinier, F. (2010) CLLD8/KMT1F is a lysine methyltransferase that is important for chromosome segregation. *J Biol Chem*, **285**, 20234-20241.
27. Ferrari, K.J., Scelfo, A., Jammula, S., Cuomo, A., Barozzi, I., Stutzer, A., Fischle, W., Bonaldi, T. and Pasini, D. (2014) Polycomb-dependent H3K27me1 and H3K27me2 regulate active transcription and enhancer fidelity. *Mol Cell*, **53**, 49-62.

28. Cao, R., Wang, L., Wang, H., Xia, L., Erdjument-Bromage, H., Tempst, P., Jones, R.S. and Zhang, Y. (2002) Role of histone H3 lysine 27 methylation in Polycomb-group silencing. *Science*, **298**, 1039-1043.
29. Margueron, R., Li, G., Sarma, K., Blais, A., Zavadil, J., Woodcock, C.L., Dynlacht, B.D. and Reinberg, D. (2008) Ezh1 and Ezh2 maintain repressive chromatin through different mechanisms. *Mol Cell*, **32**, 503-518.
30. Agger, K., Cloos, P.A., Christensen, J., Pasini, D., Rose, S., Rappsilber, J., Issaeva, I., Canaani, E., Salcini, A.E. and Helin, K. (2007) UTX and JMJD3 are histone H3K27 demethylases involved in HOX gene regulation and development. *Nature*, **449**, 731-734.
31. De Santa, F., Totaro, M.G., Prosperini, E., Notarbartolo, S., Testa, G. and Natoli, G. (2007) The histone H3 lysine-27 demethylase Jmjd3 links inflammation to inhibition of polycomb-mediated gene silencing. *Cell*, **130**, 1083-1094.
32. Walport, L.J., Hopkinson, R.J., Vollmar, M., Madden, S.K., Gileadi, C., Oppermann, U., Schofield, C.J. and Johansson, C. (2014) Human UTY(KDM6C) is a male-specific N-methyl lysyl demethylase. *J Biol Chem*, **289**, 18302-18313.
33. Mikkelsen, T.S., Ku, M., Jaffe, D.B., Issac, B., Lieberman, E., Giannoukos, G., Alvarez, P., Brockman, W., Kim, T.K., Koche, R.P. *et al.* (2007) Genome-wide maps of chromatin state in pluripotent and lineage-committed cells. *Nature*, **448**, 553-560.
34. Plath, K., Fang, J., Mlynarczyk-Evans, S.K., Cao, R., Worringer, K.A., Wang, H., de la Cruz, C.C., Otte, A.P., Panning, B. and Zhang, Y. (2003) Role of histone H3 lysine 27 methylation in X inactivation. *Science*, **300**, 131-135.
35. Wagner, E.J. and Carpenter, P.B. (2012) Understanding the language of Lys36 methylation at histone H3. *Nat Rev Mol Cell Biol*, **13**, 115-126.
36. Yuan, W., Xie, J., Long, C., Erdjument-Bromage, H., Ding, X., Zheng, Y., Tempst, P., Chen, S., Zhu, B. and Reinberg, D. (2009) Heterogeneous nuclear ribonucleoprotein L Is a subunit of human KMT3a/Set2 complex required for H3 Lys-36 trimethylation activity in vivo. *J Biol Chem*, **284**, 15701-15707.
37. Sun, X.J., Wei, J., Wu, X.Y., Hu, M., Wang, L., Wang, H.H., Zhang, Q.H., Chen, S.J., Huang, Q.H. and Chen, Z. (2005) Identification and characterization of a novel human histone H3 lysine 36-specific methyltransferase. *J Biol Chem*, **280**, 35261-35271.
38. Qiao, Q., Li, Y., Chen, Z., Wang, M., Reinberg, D. and Xu, R.M. (2011) The structure of NSD1 reveals an autoregulatory mechanism underlying histone H3K36 methylation. *J Biol Chem*, **286**, 8361-8368.
39. Li, Y., Trojer, P., Xu, C.F., Cheung, P., Kuo, A., Drury, W.J., 3rd, Qiao, Q., Neubert, T.A., Xu, R.M., Gozani, O. *et al.* (2009) The target of the NSD family of histone lysine methyltransferases depends on the nature of the substrate. *J Biol Chem*, **284**, 34283-34295.

40. Eram, M.S., Bustos, S.P., Lima-Fernandes, E., Siarheyeva, A., Senisterra, G., Hajian, T., Chau, I., Duan, S., Wu, H., Dombrovski, L. *et al.* (2014) Trimethylation of histone H3 lysine 36 by human methyltransferase PRDM9 protein. *J Biol Chem*, **289**, 12177-12188.
41. Tsukada, Y., Fang, J., Erdjument-Bromage, H., Warren, M.E., Borchers, C.H., Tempst, P. and Zhang, Y. (2006) Histone demethylation by a family of JmjC domain-containing proteins. *Nature*, **439**, 811-816.
42. He, J., Kallin, E.M., Tsukada, Y. and Zhang, Y. (2008) The H3K36 demethylase Jhdm1b/Kdm2b regulates cell proliferation and senescence through p15(Ink4b). *Nat Struct Mol Biol*, **15**, 1169-1175.
43. Weinberg, D.N., Papillon-Cavanagh, S., Chen, H., Yue, Y., Chen, X., Rajagopalan, K.N., Horth, C., McGuire, J.T., Xu, X., Nikbakht, H. *et al.* (2019) The histone mark H3K36me2 recruits DNMT3A and shapes the intergenic DNA methylation landscape. *Nature*, **573**, 281-286.
44. Carrozza, M.J., Li, B., Florens, L., Suganuma, T., Swanson, S.K., Lee, K.K., Shia, W.J., Anderson, S., Yates, J., Washburn, M.P. *et al.* (2005) Histone H3 methylation by Set2 directs deacetylation of coding regions by Rpd3S to suppress spurious intragenic transcription. *Cell*, **123**, 581-592.
45. Fnu, S., Williamson, E.A., De Haro, L.P., Brenneman, M., Wray, J., Shaheen, M., Radhakrishnan, K., Lee, S.H., Nickoloff, J.A. and Hromas, R. (2011) Methylation of histone H3 lysine 36 enhances DNA repair by nonhomologous end-joining. *Proc Natl Acad Sci U S A*, **108**, 540-545.
46. An, S., Yeo, K.J., Jeon, Y.H. and Song, J.J. (2011) Crystal structure of the human histone methyltransferase ASH1L catalytic domain and its implications for the regulatory mechanism. *J Biol Chem*, **286**, 8369-8374.
47. Rahman, S., Sowa, M.E., Ottinger, M., Smith, J.A., Shi, Y., Harper, J.W. and Howley, P.M. (2011) The Brd4 extraterminal domain confers transcription activation independent of pTEFb by recruiting multiple proteins, including NSD3. *Mol Cell Biol*, **31**, 2641-2652.
48. Brown, M.A., Sims, R.J., 3rd, Gottlieb, P.D. and Tucker, P.W. (2006) Identification and characterization of Smyd2: a split SET/MYND domain-containing histone H3 lysine 36-specific methyltransferase that interacts with the Sin3 histone deacetylase complex. *Mol Cancer*, **5**, 26.
49. Eom, G.H., Kim, K.B., Kim, J.H., Kim, J.Y., Kim, J.R., Kee, H.J., Kim, D.W., Choe, N., Park, H.J., Son, H.J. *et al.* (2011) Histone methyltransferase SETD3 regulates muscle differentiation. *J Biol Chem*, **286**, 34733-34742.
50. Lee, S.H., Oshige, M., Durant, S.T., Rasila, K.K., Williamson, E.A., Ramsey, H., Kwan, L., Nickoloff, J.A. and Hromas, R. (2005) The SET domain protein Metnase mediates foreign DNA integration and links integration to nonhomologous end-joining repair. *Proc Natl Acad Sci U S A*, **102**, 18075-18080.

51. Jack, A.P., Bussemer, S., Hahn, M., Punzeler, S., Snyder, M., Wells, M., Csankovszki, G., Solovei, I., Schotta, G. and Hake, S.B. (2013) H3K56me3 is a novel, conserved heterochromatic mark that largely but not completely overlaps with H3K9me3 in both regulation and localization. *PLoS One*, **8**, e51765.
52. Daujat, S., Weiss, T., Mohn, F., Lange, U.C., Ziegler-Birling, C., Zeissler, U., Lappe, M., Schubeler, D., Torres-Padilla, M.E. and Schneider, R. (2009) H3K64 trimethylation marks heterochromatin and is dynamically remodeled during developmental reprogramming. *Nat Struct Mol Biol*, **16**, 777-781.
53. Lange, U.C., Siebert, S., Wossidlo, M., Weiss, T., Ziegler-Birling, C., Walter, J., Torres-Padilla, M.E., Daujat, S. and Schneider, R. (2013) Dissecting the role of H3K64me3 in mouse pericentromeric heterochromatin. *Nat Commun*, **4**, 2233.
54. Kirmizis, A., Santos-Rosa, H., Penkett, C.J., Singer, M.A., Vermeulen, M., Mann, M., Bahler, J., Green, R.D. and Kouzarides, T. (2007) Arginine methylation at histone H3R2 controls deposition of H3K4 trimethylation. *Nature*, **449**, 928-932.
55. Kim, S., Kim, N.H., Park, J.E., Hwang, J.W., Myung, N., Hwang, K.T., Kim, Y.A., Jang, C.Y. and Kim, Y.K. (2020) PRMT6-mediated H3R2me2a guides Aurora B to chromosome arms for proper chromosome segregation. *Nat Commun*, **11**, 612.
56. Guccione, E., Bassi, C., Casadio, F., Martinato, F., Cesaroni, M., Schuchlautz, H., Luscher, B. and Amati, B. (2007) Methylation of histone H3R2 by PRMT6 and H3K4 by an MLL complex are mutually exclusive. *Nature*, **449**, 933-937.
57. Walport, L.J., Hopkinson, R.J., Chowdhury, R., Schiller, R., Ge, W., Kawamura, A. and Schofield, C.J. (2016) Arginine demethylation is catalysed by a subset of JmjC histone lysine demethylases. *Nat Commun*, **7**, 11974.
58. Chang, B., Chen, Y., Zhao, Y. and Bruick, R.K. (2007) JMJD6 is a histone arginine demethylase. *Science*, **318**, 444-447.
59. Blythe, S.A., Cha, S.W., Tadjuidje, E., Heasman, J. and Klein, P.S. (2010) beta-Catenin primes organizer gene expression by recruiting a histone H3 arginine 8 methyltransferase, Prmt2. *Dev Cell*, **19**, 220-231.
60. Wang, L., Pal, S. and Sif, S. (2008) Protein arginine methyltransferase 5 suppresses the transcription of the RB family of tumor suppressors in leukemia and lymphoma cells. *Mol Cell Biol*, **28**, 6262-6277.
61. Pal, S., Vishwanath, S.N., Erdjument-Bromage, H., Tempst, P. and Sif, S. (2004) Human SWI/SNF-associated PRMT5 methylates histone H3 arginine 8 and negatively regulates expression of ST7 and NM23 tumor suppressor genes. *Mol Cell Biol*, **24**, 9630-9645.
62. Botuyan, M.V., Lee, J., Ward, I.M., Kim, J.E., Thompson, J.R., Chen, J. and Mer, G. (2006) Structural basis for the methylation state-specific recognition of histone H4-K20 by 53BP1 and Crb2 in DNA repair. *Cell*, **127**, 1361-1373.

63. Kuo, A.J., Song, J., Cheung, P., Ishibe-Murakami, S., Yamazoe, S., Chen, J.K., Patel, D.J. and Gozani, O. (2012) The BAH domain of ORC1 links H4K20me2 to DNA replication licensing and Meier-Gorlin syndrome. *Nature*, **484**, 115-119.
64. Schotta, G., Lachner, M., Sarma, K., Ebert, A., Sengupta, R., Reuter, G., Reinberg, D. and Jenuwein, T. (2004) A silencing pathway to induce H3-K9 and H4-K20 trimethylation at constitutive heterochromatin. *Genes Dev*, **18**, 1251-1262.
65. Schotta, G., Sengupta, R., Kubicek, S., Malin, S., Kauer, M., Callen, E., Celeste, A., Pagani, M., Opravil, S., De La Rosa-Velazquez, I.A. *et al.* (2008) A chromatin-wide transition to H4K20 monomethylation impairs genome integrity and programmed DNA rearrangements in the mouse. *Genes Dev*, **22**, 2048-2061.
66. Yang, H., Pesavento, J.J., Starnes, T.W., Cryderman, D.E., Wallrath, L.L., Kelleher, N.L. and Mizzen, C.A. (2008) Preferential dimethylation of histone H4 lysine 20 by Suv4-20. *J Biol Chem*, **283**, 12085-12092.
67. Wang, J., Telese, F., Tan, Y., Li, W., Jin, C., He, X., Basnet, H., Ma, Q., Merkurjev, D., Zhu, X. *et al.* (2015) LSD1n is an H4K20 demethylase regulating memory formation via transcriptional elongation control. *Nat Neurosci*, **18**, 1256-1264.
68. Brejc, K., Bian, Q., Uzawa, S., Wheeler, B.S., Anderson, E.C., King, D.S., Kranzusch, P.J., Preston, C.G. and Meyer, B.J. (2017) Dynamic Control of X Chromosome Conformation and Repression by a Histone H4K20 Demethylase. *Cell*, **171**, 85-102 e123.
69. Xu, J. and Kidder, B.L. (2018) H4K20me3 co-localizes with activating histone modifications at transcriptionally dynamic regions in embryonic stem cells. *BMC Genomics*, **19**, 514.
70. Stender, J.D., Pascual, G., Liu, W., Kaikkonen, M.U., Do, K., Spann, N.J., Boutros, M., Perrimon, N., Rosenfeld, M.G. and Glass, C.K. (2012) Control of proinflammatory gene programs by regulated trimethylation and demethylation of histone H4K20. *Mol Cell*, **48**, 28-38.
71. Li, S., Ali, S., Duan, X., Liu, S., Du, J., Liu, C., Dai, H., Zhou, M., Zhou, L., Yang, L. *et al.* (2018) JMJD1B Demethylates H4R3me2s and H3K9me2 to Facilitate Gene Expression for Development of Hematopoietic Stem and Progenitor Cells. *Cell Rep*, **23**, 389-403.
72. Zhao, Q., Rank, G., Tan, Y.T., Li, H., Moritz, R.L., Simpson, R.J., Cerruti, L., Curtis, D.J., Patel, D.J., Allis, C.D. *et al.* (2009) PRMT5-mediated methylation of histone H4R3 recruits DNMT3A, coupling histone and DNA methylation in gene silencing. *Nat Struct Mol Biol*, **16**, 304-311.
